# A higher-level MRP supertree of placental mammals

**DOI:** 10.1186/1471-2148-6-93

**Published:** 2006-11-13

**Authors:** Robin MD Beck, Olaf RP Bininda-Emonds, Marcel Cardillo, Fu-Guo Robert Liu, Andy Purvis

**Affiliations:** 1 Division of Biology, Imperial College London, Silwood Park campus, Ascot SL5 7PY, UK; 2 Natural History Museum, Cromwell Road, London SW7 5BD, UK; 3 School of Biological, Earth and Environmental Sciences, University of New South Wales, NSW 2052, Australia; 4 Lehrstuhl für Tierzucht, Technical University of Munich, 85354 Freising-Weihenstephan, Germany; 5 Institut für Spezielle Zoologie und Evolutionsbiologie mit Phyletischem Museum, Friedrich-Schiller-Universität Jena, 07743 Jena, Germany; 6 Department of Zoology, Box 118525, University of Florida, Gainesville, Florida 32611-8552, USA

## Abstract

**Background:**

The higher-level phylogeny of placental mammals has long been a phylogenetic Gordian knot, with disagreement about both the precise contents of, and relationships between, the extant orders. A recent MRP supertree that favoured 'outdated' hypotheses (notably, monophyly of both Artiodactyla and Lipotyphla) has been heavily criticised for including low-quality and redundant data. We apply a stringent data selection protocol designed to minimise these problems to a much-expanded data set of morphological, molecular and combined source trees, to produce a supertree that includes every family of extant placental mammals.

**Results:**

The supertree is well-resolved and supports both polyphyly of Lipotyphla and paraphyly of Artiodactyla with respect to Cetacea. The existence of four 'superorders' – Afrotheria, Xenarthra, Laurasiatheria and Euarchontoglires – is also supported. The topology is highly congruent with recent (molecular) phylogenetic analyses of placental mammals, but is considerably more comprehensive, being the first phylogeny to include all 113 extant families without making *a priori *assumptions of suprafamilial monophyly. Subsidiary analyses reveal that the data selection protocol played a key role in the major changes relative to a previously published higher-level supertree of placentals.

**Conclusion:**

The supertree should provide a useful framework for hypothesis testing in phylogenetic comparative biology, and supports the idea that biogeography has played a crucial role in the evolution of placental mammals. Our results demonstrate the importance of minimising poor and redundant data when constructing supertrees.

## Background

The higher-level phylogeny of placental mammals has long been one of the most intensively studied problems in systematics (e.g [1-6]), because a robust placental phylogeny is crucial to understanding mammalian evolution and biogeography. Until relatively recently, most comprehensive studies have relied purely on morphological data. Such studies largely upheld the monophyly of all 18 [7] traditionally recognised orders but were rather less successful in resolving the relationships between the orders (e.g. [4]).

Recent sophisticated analyses of molecular sequence data have significantly revised and refined the view from morphology, resulting in a well-resolved 'molecular consensus' view of placental phylogeny [8,9] that is broadly supported by many genes and gene combinations (see Table 1). This consensus rejects the monophyly of two traditional placental orders: Artiodactyla (even-toed 'ungulates') is paraphyletic with respect to Cetacea (whales; [10,11]) and Lipotyphla (the 'insectivores') is diphyletic [12,13], being split into Afrosoricida and Eulipotyphla. At the interordinal level, molecular data consistently resolve extant placental groups into four 'superorders': Afrotheria, Xenarthra, Laurasiatheria and Euarchontoglires (the latter two comprising Boreoeutheria). Despite this recent progress, regions of the topology are still uncertain, as different data types (e.g. nuclear genes, mitochondrial genes, morphology) and methods of analysis (e.g. maximum parsimony, maximum likelihood) often support conflicting relationships. Notably, the location of the root of the placental tree remains unresolved [8,14-16], and the precise relationships within each superorder are also somewhat unclear. Perhaps more importantly, taxonomic coverage remains far from complete: the taxonomically most inclusive higher-level single-matrix analysis of mammals so far, that of Murphy et al. [17], included representatives of only 54 of the 113 extant placental families recognised by Wilson and Reeder [7]. Studies directly combining molecular and morphological data have been even more taxonomically limited, tending to focus on specific areas of contention, such as afrotherian monophyly [18], or relationships within Cetartiodactyla [11]. This is because comprehensiveness in such analyses is difficult to achieve, given the typically patchy taxonomic distribution of available data [19,20] that can be analysed in a single matrix.

**Table 1:** Superorders and selected additional supraordinal clades currently supported by the 'molecular consensus' view of placental phylogeny (e.g. [8,9]).

**Superorders**	**Supraordinal clades**	**Orders**	**Common names of orders**
Afrotheria	Paenungulata	Hyracoidea	Hyraxes
		Proboscidea	Elephants
		Sirenia	Seacows
	Afroinsectiphilia	Afrosoricida	African 'insectivores' (tenrecs and golden moles)
		Macroscelidea	Elephant shrews
		Tubulidentata	Aardvark

Xenarthra		Cingulata	Armadillos
		Pilosa	Anteaters and sloths

Euarchontoglires	Glires	Lagomorpha	Lagomorphs
		Rodentia	Rodents
	Euarchonta	Dermoptera	Flying lemurs
		Primates	Primates
		Scandentia	Tree shrews

Laurasiatheria		Eulipotyphla	True 'insectivores' (hedgehogs, shrews, true moles and *Solenodon*)
	Fereuungulata	Carnivora	Carnivorans
		Cetartiodactyla	Even-toed 'ungulates' and whales
		Chiroptera	Bats
		Perissodactyla	Odd-toed 'ungulates'
		Pholidota	Pangolins

Orders follow [7] except that Xenarthra is divided into Cingulata and Pilosa, Lipotyphla ('insectivores') is split between Afrosoricida and Eulipotyphla, and Cetacea (whales) and Artiodactyla (even-toed 'ungulates) are combined as Cetartiodactyla. Higher-level names are taken from [44].

Supertree analysis provides an alternative route to comprehensive estimates of phylogeny [21]. This approach combines existing phylogenetic tree topologies ('source trees'), rather than their underlying data, by any of a number of methods – most commonly Matrix Representation with Parsimony (MRP; [22,23]). This procedure produces a composite phylogeny, or 'supertree', that can be taxonomically more comprehensive than any source tree. Because supertree analyses sample at the level of tree topologies [24], source trees based on any data (e.g. distances, which cannot be incorporated into ordinary phylogenetic character matrices), can be used. As a result, supertrees can be based on the broadest sampling of both data and taxa, and so are often taxonomically more comprehensive than phylogenies of the same clades produced by more direct approaches (e.g. [25-27]). Supertrees of many clades have now been published, almost exclusively using MRP (see [28] for a recent review).

Liu et al. ([29]; henceforth 'LEA') used a supertree approach to infer the relationships among placental mammal families from a combination of morphological and molecular source trees. Their combined supertree, based on 430 source trees from 315 references published before March 1999, still remains by far the most comprehensive higher-level phylogeny of placentals published.

Overall, the LEA combined supertree ([29]; their Fig. 1) seemed a reasonable compromise between the morphological and molecular phylogenies then available [29]. However, it conflicted with the majority of more recent data in parts of its topology, supporting instead 'outdated' views of placental phylogeny (see [11]). Most notably, Artiodactyla was monophyletic, contradicting a wealth of evidence already then available (and subsequently greatly reinforced) for a Cetacea + Hippopotamidae (hippos) clade (= Whippomorpha; summarised in [11]). Furthermore, interfamilial relationships within Artiodactyla appeared anomalous [11]. Monophyly of Lipotyphla was also strongly supported, contradicting the association between Afrosoricida and the other taxa (Paenungulata, Macroscelidea and Tubulidentata) now considered to comprise Afrotheria [30]. This was despite considerable molecular evidence for both lipotyphlan polyphyly and afrotherian monophyly prior to March 1999 (e.g. [12,30]), both of which were actually reflected in the molecular-only supertree of LEA ([29]; their Fig. 2A).

**Figure 1:**
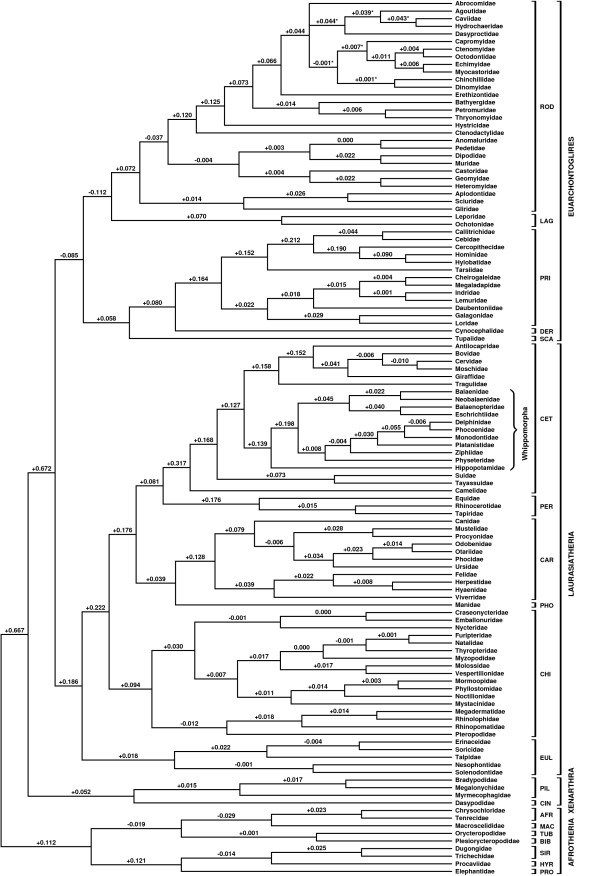
**Supertree of extant placentals (50% majority rule consensus of 17 most parsimonious trees – length = 8150.935), following application of the protocol of Bininda-Emonds et al. [32] to the complete set of references**. Asterisks indicate which branches collapse in the strict consensus. Numbers above branches represent reduced qualitative support (rQS; [26,39]) values. The orders are indicated by brackets and the first three letters of their names following Table 1, with the additional fossil order Bibymalagasia indicated by BIB. Whippomorpha and the four superorders are also indicated.

**Figure 2:**
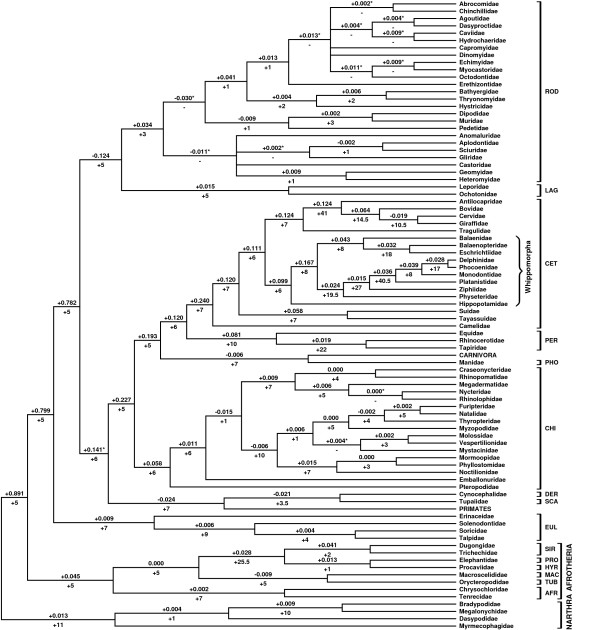
**Supertree of extant placentals (50% majority rule consensus of 5540 most parsimonious trees – length = 4262.625), following application of the protocol of Bininda-Emonds et al. [32] to only those references used by Liu et al. [29]**. Asterisks indicate which branches collapse in the strict consensus. Numbers above branches represent rQS values, and numbers below represent decay indices. The orders and Whippomorpha are indicated and bracketed as in Figure 1, as are the two superorders (Xenarthra and Afrotheria) recovered as monophyletic in this analysis.

Gatesy et al. [11] argued in detail that the 'outdated' features of the LEA supertree stemmed from any or all of: 1) uncritical selection of source trees that represent poor and duplicated data; 2) assumptions of ordinal monophyly without basis in the underlying data ('appeals to authority'); and 3) inherent, methodological shortcomings in the MRP method, if not the supertree approach as a whole (see also [31]). Concentrating on the relationship between Artiodactyla and Cetacea, Gatesy et al. [11] claimed that *all *of the 33 MRP pseudocharacters supporting the monophyly of Artiodactyla in the combined supertree derived from low quality source trees that represented 'appeals to authority, duplications of data, miscodings, or derivatives of poorly justified trees' [11].

Motivated by concerns about source tree quality and duplication in supertree analyses, Bininda-Emonds et al. ([32]; summarised in [33]) proposed a set of guidelines for identifying suitable source trees, filtering out trees representing duplicated or poor data, and minimizing assumptions of higher-taxon monophyly. The underlying principle of the guidelines is that only those source trees that can be considered to represent 'independent phylogenetic hypotheses' should be included in a supertree. Bininda-Emonds et al. [32] proposed that source trees produced from independent character sets, such as different genes or different morphological character sets, all represent such independent phylogenetic hypotheses. They also contended that different combinations of genes and/or morphological characters likewise comprise independent phylogenetic hypotheses, because of the possibility of signal enhancement (*sensu *[34]). To minimise data duplication, they suggested that, where no clear cut choice for a single tree presents itself for a given independent character set (e.g. a particular gene), MRP 'mini-supertrees' of all non-independent source trees based on that character set should be created. Thus, each dataset adjudged to be independent according the protocol is ultimately represented (as far as possible) by a single, taxonomically inclusive tree – either an original source tree or a 'mini-supertree' – in the final supertree analysis. This protocol has already been followed in the construction of species-level supertrees of extant marsupials [25] and cetartiodactyls [26].

Here, we apply the Bininda-Emonds et al. [32] guidelines to a large set of source trees, including all those used by LEA but also those from references published between March 1999 (LEA's cut-off date) and September 2004, to investigate higher-level placental phylogeny. We include all 113 extant placental families, plus two recently extinct and enigmatic groups – Nesophontidae (West Indian shrew-like 'insectivores', currently included in Lipotyphla; [7]) and Plesiorycteropodidae (a myrmecophagous form recently assigned its own order, Bibymalagasia; [35]) – the relationships of which may be crucial to a better understanding of both the biogeographical history and patterns of character evolution within placentals [36]. We use a modified, 'semi-rooted' version of MRP that can compensate for source trees that are not robustly rooted [37]. We assess the degree of support for nodes in the supertree using a supertree-specific support measure, reduced qualitative support (rQS; [26]); this varies from -1 (no support) to +1 (support from all relevant source trees), and is described in Methods.

As a subsidiary analysis, we apply the guidelines of Bininda-Emonds et al. [32] to the same 315 references used by LEA in their combined supertree. By reproducing their methodology as far as possible (e.g. exclusion of specific taxa, weighting of specific source trees, use of standard rooted MRP coding), except where these conflict with the recommendations of the protocol, we aim to assess the specific impact of the protocol on the overall supertree topology. Specifically, we focus on whether monophyly of both Artiodactyla and Lipotyphla are affected in terms of changes in topology, or in support values as measured by the decay index (DI; [38]). We also examine whether other changes in topology and support are in better or worse agreement with contemporary evidence. This will help determine whether the criticized aspects of the original LEA topology reflect an inherent, unavoidable, weakness of supertree analysis *per se*, or avoidable weaknesses in the source dataset that was originally used and that can be remedied using a suitable protocol for source tree collection.

## Results and discussion

The search of the final MRP supertree matrix from the full analysis recovered 17 most parsimonious trees of length 8150.935 (the tree length is not a whole number because of the downweighting procedure used to account for the presence of nonmonophyletic families in some source trees; see Methods). The strict consensus of the 17 trees is highly resolved, with the only conflict occurring within the hystricognath rodents. The 50% majority rule consensus is illustrated here (Figure 1), with those branches that collapse in the strict consensus identified by asterisks. There are no unsupported novel clades (*sensu *[39]). Repeating the analysis with the extinct Nesophontidae and Plesiorycteropodidae deleted from the original source trees has no effect on the higher-level relationships among the extant taxa.

The supertree presented here is highly congruent with most recent estimates of placental phylogeny at both the inter- and intraordinal levels [8,9]. However, because the primary goal of our analyses was to investigate ordinal composition and interordinal relationships, we did not include single order source trees in our dataset. As such, the intraordinal relationships presented here are not based on the maximum amount of data available. Although even this amount of data has yielded relationships that are largely congruent with current phylogenetic opinion, we would recommend the use of relevant supertrees (e.g. [26,27]) or other similarly comprehensive phylogenies for intraordinal relationships.

Four principal clades, or 'superorders', are present: Afrotheria (rQS = +0.112), Xenarthra (rQS = +0.052), Laurasiatheria (rQS = +0.186) and Euarchontoglires (rQS = -0.085). In upholding the monophyly of these superorders, this supertree supports the hypothesis that plate tectonics have been crucial in the early evolution of modern placentals [40]. The superorders may have undergone their initial divergences in biogeographical areas that were separate throughout much of the Cretaceous and Cenozoic: Afrotheria in Afro-Arabia, Xenarthra in South America, and both Euarchontoglires and Laurasiatheria in Laurasia [40]. However, recent studies suggest that a number of fossil 'condylarths' from Laurasia are afrotherian [18,41], conflicting with a strict tectonic-based interpretation of placental phylogeny. Regardless, these four superorders indicate that morphological convergence has been more pervasive than previously thought [8,36,42].

In agreement with most recent phylogenetic analyses of placental mammals, the supertree upholds the monophyly of 16 of the 18 extant orders recognised in Wilson and Reeder [7]. Although a 'seed tree' that assumes monophyly of all 18 of these orders (including Artiodactyla and Lipotyphla) was included as a source tree (see Methods), the 16 that are monophyletic in the supertree are supported by between 17 (Sirenia) and 156 (Primates) other source trees. Lipotyphla is polyphyletic, with Afrosoricida in Afrotheria and Eulipotyphla (here including the extinct Nesophontidae) in Laurasiatheria, and Artiodactyla is paraphyletic with respect to Cetacea.

The supertree supports Afrotheria as the sister to the remaining superorders, in agreement with most nuclear and nuclear + mitochondrial trees (e.g. [43-45]). A recent analysis of retroposon integrations [16] supported a xenarthran root, congruent with morphological evidence for a split between Xenarthra and all other extant placentals (Epitheria) [46], although alternatives could not be statistically rejected. Within Afrotheria, the first split is between Paenungulata (rQS = +0.121) and Afroinsectiphilia (rQS = -0.019; here including the extinct Plesiorycteropodidae). Within Paenungulata, Procaviidae (Hyracoidea) and Dugongidae + Trichechidae (Sirenia) are sister taxa (rQS = -0.014), in agreement with some sequence data (e.g. [13,43]) and retroposons [47]. Within Afroinsectiphilia, the supertree recovers both Afrosoricida (rQS = +0.023) and Afroinsectivora (Afrosoricida + Macroscelididae; rQS = -0.029), with Orycteropodidae (Tubulidentata) as the sister to Afroinsectivora; this is again congruent with most sequence data (e.g. [8]), although chromosome-painting supports monophyly of (Macroscelididae + Orycteropodidae) [48] and retroposons support monophyly of (Afrosoricida + Orycteropodidae) [47]. Based on source trees from MacPhee [35] and Asher *et al*. [18], Plesiorycteropodidae is recovered as the sister to Orycteropodidae, indicating that the extinct bibymalagasy is afrotherian, as might be suspected from its known distribution (the Holocene of Madagascar; [35]) and from features of its astragalus that are shared with a number of extant afrotherians [35,36]. Relationships within Xenarthra, the only superorder that is currently also supported by morphology, are congruent with both morphological [49] and molecular [14] evidence.

Euarchonta (rQS = +0.058) and Glires (rQS = -0.112) are both monophyletic, together forming the clade Euarchontoglires. The low rQS value for Glires probably reflects the inclusion of source trees that support rodent polyphyly or paraphyly (e.g. [50,51]), although morphological [18] and most recent molecular phylogenies [17,43,52] support rodent monophyly, as recovered here. Tupaiidae (Scandentia) form the sister group to a Cynocephalidae (Dermoptera) + Primates clade. The supertree topology within Euarchontoglires, at both the inter- and intra-ordinal levels, is highly congruent with most recent, mainly molecular evidence [17,43,44,52].

Within Laurasiatheria, a monophyletic Eulipotyphla (rQS = +0.018) is the sister to the remaining taxa. This contradicts the hypothesis that Erinaceidae (hedgehogs) are basal placentals, as has been suggested by mitochondrial trees (e.g. [50]),. A Solenodontidae + Nesophontidae (rQS = -0.001) clade is congruent with biogeographic evidence, as both taxa are known only from the West Indies, but compelling evidence for the true affinities of Nesophontidae is still lacking [36]; the position advocated for it here is based on only three source trees. A sister-group relationship between Erinaceidae and Soricidae (shrews) to the exclusion of Talpidae (true moles) agrees with most molecular estimates (e.g. [53]), but is only relatively weakly supported here (rQS = -0.004). Within the remaining taxa, Chiroptera (including a paraphyletic Microchiroptera with respect to Megachiroptera) are the sister group to Fereuungulata (rQS = +0.176). Carnivora and Manidae (Pholidota) together form Ferae (rQS = +0.039), with Cetartiodactyla and Perissodactyla as sister taxa (= Cetungulata; rQS = +0.081). Different molecular data continue to yield incompatible topologies within Fereuungulata (see [44]); the topology favoured here is arguably more congruent with morphological data because the sister relationship between cetartiodactyls and perissodactyls requires only a single origin of 'ungulate' features within Laurasiatheria. However, a recent transposon analysis [54] recovered a clade comprising carnivorans, perissodactyls and bats (pholidotans were not sampled, but are also probably members of this group), which has been named Pegasoferae. Artiodactyla is paraphyletic, with Whippomorpha (rQS = +0.139) as the sister to the ruminants, forming Cetruminantia (rQS = +0.127); Suidae + Tayassuidae (pigs + peccaries; rQS = +0.073) and Camelidae (camels) comprise successive sister groups. The cetartiodactylan topology is congruent with both molecular [10] and combined morphological and molecular [11] data.

Overall, our supertree topology is in much better agreement with the current consensus view of placental phylogeny than is that of LEA. Why? The three main possibilities are (a) that the LEA topology resulted from either poor and/or duplicated data, or assumptions of monophyly, which the Bininda-Emonds et al. [32] guidelines have largely removed; (b) that other, minor, differences in the technical details of the two studies are responsible, or (c) that phylogenies published after March 1999 (the cut-off point of LEA) are more in agreement with the molecular consensus, and that these studies are now in a majority. Our subsidiary analysis – in effect, repeating the LEA analysis using the Bininda-Emonds et al. [32] guidelines – can help discriminate between these three possibilities.

The subsidiary analysis found 5535 trees of length 4262.625, using the same 4:1 weighting scheme of LEA (see Methods). A strict consensus is fully resolved at the interordinal level (the only conflicts are within rodents and bats), and there are no novel unsupported clades. Again, although a 'seed tree' that assumes monophyly of the orders recognised by Wilson and Reeder [7] was included as a source tree (see Methods), those that are monophyletic in the supertree are supported by between six (Sirenia) and 60 (Rodentia) other source trees. Figure 2 is a 50% majority rule consensus, with branches that collapse in the strict consensus indicated by asterisks. The equally weighted analysis (not shown) recovers largely identical unrooted relationships, with neither Artiodactyla nor Lipotyphla recovered as monophyletic.

Artiodactyla is paraphyletic, with Cetacea and Hippopotamidae forming Whippomorpha. Support values (DI = 6; rQS = +0.099) indicate that this clade is relatively well-supported, and are similar to those for Ruminantia (DI = 7; rQS = +0.124). Whippomorpha and Ruminantia are sisters, forming Cetruminantia, which also has reasonable support (DI = 6; rQS = +0.111).

Lipotyphla is polyphyletic, with separate eulipotyphlan (DI = 7, rQS = +0.009) and afrosoricid (DI = 7, rQS = +0.002) clades. Significantly, Afrosoricida is part of a monophyletic Afrotheria (DI = 5, rQS = +0.045), the existence of which was controversial in 1999. Afrotheria was not recovered in the combined supertree of LEA, although it was present in their molecular-only supertree (their Figure 2A).

The two major changes from the LEA topology – Cetacea nesting within a paraphyletic Artiodactyla, and diphyly of Lipotyphla (both of which were recovered in the LEA molecular-only supertree) – seen in this reanalysis are both in better accord with the state of phylogenetic knowledge in 1999, and are in agreement with our full supertree (Figure 1). They indicate that the potential problem of 'time-lag' in supertrees, where inclusion of older studies biases the supertree topology towards outdated views of relationships, is not an inherent limitation of the method.

Notably, the DI support values in this reanalysis are almost always lower, and in many cases greatly so, than their equivalents in the original combined LEA supertree. For example, DI support for the monophyly of the order Chiroptera, drops from 74 to 6, and similarly large drops are seen for Lagomorpha (69 to 5), Perissodactyla (139 to 10) and Rodentia (37 to 3). Some interordinal groupings also show reduced DI values (e.g. Glires, 26 to 5; Ferae, 21 to 7; Paenungulata, 108 to 25.5). These declines probably reflect the exclusion of some duplicate trees and, particularly, the avoidance of *a priori *assumptions of monophyly. As such, the DI values in this analysis are probably a more accurate indication of the actual support for each group.

Table 2 lists the relative similarity of different topologies as measured by the normalised partition metric [55,56] and 'explicitly agree' triplets. It indicates that both the application of the source tree selection protocol of Bininda-Emonds et al. [32] and the inclusion of more recent source trees are important in explaining the differences between our updated supertree topology and the original LEA supertree. For instance, the 4:1 upweighted supertree from the subsidiary analysis ('LEA+P 4:1') is ~18% more similar to the large molecular tree of Murphy et al. ('MEA'; [17]) than is the original LEA supertree, according to the normalised partition metric. This effect is attributable solely to the application of the protocol, which was sufficient to bring the LEA dataset in line with the molecular consensus in a number of key areas. The full supertree, however, was ~15% more similar again to the Murphy et al. [17] tree. It is also only ~9% different from the large molecular and morphological analysis of Gatesy et al. ('GEA'; [11]). These latter results reflect the inclusion of the more recent source trees published since the study by LEA. The 'explicitly agree' triplet scores confirm these findings.

**Table 2:** Normalised partition metric [55,56] and 'explicitly agree' triplet scores of supertrees and supermatrices.

		Normalised partition metric					
		**LEA**	**LEA+P 1:1**	**LEA+P 4:1**	**Full ST**	**MEA**	**GEA**
'Explicitly agree triplets'	**LEA**	-	0.327	0.244	0.409	0.464	0.179
	**LEA+P 1:1**	0.241	-	0.143	0.305	0.345	0.185
	**LEA+P 4:1**	0.021	0.227	-	0.317	0.286	0.143
	**Full ST**	0.119	0.313	0.102	-	0.135	0.091
	**MEA**	0.168	0.457	0.146	0.001	-	0.105
	**GEA**	0.054	0.080	0.008	0.005	0.006	-

'LEA' = Combined morphological and molecular supertree of Liu et al. ([29]; their Figure 1); 'LEA+P 1:1' = 1:1 equally weighted supertree following application of protocol to the LEA references (50% majority rule consensus; not shown); 'LEA+P 4:1' = 4: 1 upweighted supertree following application of protocol to the LEA references (50% majority rule consensus; Figure 2); 'Full ST' = extended analysis supertree, based on an updated set of references (50% majority rule consensus; Figure 1); 'MEA' = molecular topology of Murphy et al. ([17] their Figure 1); 'GEA' = morphological and molecular topology of Gatesy et al. ([11]; their Figure 4).

## Conclusion

The supertree from our main analysis is a well-resolved, comprehensive, and reasonably robust higher-level phylogeny of placental mammals. It agrees strongly with the weight of current data (e.g. [11,17,18,42,43,45]), suggesting that MRP supertrees can accurately reflect available phylogenetic evidence (*contra *[11]). To our knowledge, it is the first placental phylogeny of any kind to include all extant families, and has over two times the taxonomic coverage of the most comprehensive non-supertree analysis so far [17].

The supertree is based on a large set of stringently-selected source trees derived from analyses of a very wide range of characters and character types (including morphology, mitochondrial genes and nuclear genes) analysed using improved coding [32,37], searching [57] and robustness-checking [26] methods from those used in the previous supertree assessment of placental phylogeny by LEA. It appears from our subsidiary analysis that at least some of the key differences between our supertree and the original LEA study lie with the selection of independent source trees and in the avoidance of *a priori *assumptions of monophyly. This finding confirms that the inclusion of poor or duplicated data is not inherent to supertree construction (as implied by [11]; see [33]), although, as in all areas of science, it remains an issue of which researchers need to be mindful.

The supertree hopefully provides a valuable, comprehensive framework for research into the evolution and biogeography of placental mammals. We suggest that this topology is suitable for use in comparative studies that require a higher-level phylogeny of placentals. Supertrees, if carefully constructed, can combine apparent accuracy (as judged by available character evidence) with comprehensiveness, suggesting that they may play an important role in phylogenetics for some time to come.

## Methods

### Finding and Filtering Source Trees

The 315 references used by LEA are listed online as supplementary information to their paper [58]. To identify additional relevant references that might contain further source trees, we searched BioAbstracts, Web of Knowledge, Zoological Record and BIOSIS online literature databases using the following search terms: mammal*, euther* or placental* together with any of phylogen*, systematic*, cladistic*, classif*, taxonom*, cladogram*, phenogram* or fossil*. We examined the online abstracts (where available) of the ~3000 initial references identified, and excluded those that did not appear to contain relevant phylogenetic information. The remaining ~1000 (including supplementary information such as electronic appendices) were examined in full, as were all of the LEA references.

We rejected potential source trees for any of several possible reasons. Trees that did not provide unequivocal evidence that actual datasets underlie their topologies (e.g., many reviews, taxonomies and informal composites of existing phylogenies) were rejected; we considered unequivocal evidence to include character lists, apomorphy lists, sequence alignments, character matrices or distance matrices. Trees reproduced from earlier references (and thus dissociated from their underlying datasets) were also excluded, although we examined the original references where possible. Source trees in which characters were mapped onto an independent topology were rejected, unless the authors demonstrated that the distribution of the mapped characters was congruent with the assumed tree. References lacking phylogeny depictions and not providing sufficient information in the text to infer a reasonably well-resolved source tree were not used, nor were those that included only unrooted trees, unless the presence of non-placental taxa or clearly identified paralogous genes made rooting uncontroversial. References containing only source trees whose terminal taxa could not be identified to the family-level or below – for example morphological studies where taxa are not identified beyond the ordinal level, or molecular studies that employ interfamilial chimeric sequences (see [59]) – were also not used. LEA coded such trees with each order replaced by an unresolved polytomy comprising its constituent families, but because the composition of the currently recognised placental orders (Lipotyphla and Artiodactyla, in particular) is in question, in addition to their interrelationships, we considered it necessary to exclude trees that would have forced us to assume ordinal monophyly *a priori*. Source trees that included some terminal taxa that were above the family-level, but that were otherwise suitable for inclusion in the final supertree, were coded with the suprafamilial taxa deleted. Because our focus is on interordinal relationships, in general we only coded additional source trees for the full analysis that included representatives of at least three placental orders recognised by Wilson and Reeder [7]. Exceptions to this were artiodactyl-only, lipotyphlan-only and rodent-only trees (with representatives from at least three families), all of which were coded because the monophyly of each of these orders has been seriously challenged in recent years ([11,18,50] respectively). The number of taxa present in each source tree varied between three and 55.

This initial filtering rejected 93 of the references originally used by LEA, leaving 222 for reanalysis. These comprised the complete set of initial source trees for our subsidiary analysis (see below). Trees from 208 further publications also met the filtering criteria. The topologies of all suitable trees presented in these 430 references [see Additional file 1] – such as multiple most parsimonious cladograms and/or trees produced under different phylogenetic methods (e.g. parsimony, distance and likelihood) and weighting schemes [see Additional file 2] – constitute the data set for our full analysis. They were reproduced by importing an appropriate taxon list into TreeView 1.6.6 [60], changing the resultant 'bush' to the appropriate topology based on all relevant information present as diagrams, tables and accompanying text (where sufficient to imply an informative phylogeny), and saving it as a NEXUS-formatted treefile [61]. We always chose the optimal trees (or consensus thereof), where indicated, over constrained or suboptimal trees preferred by the authors based on *a priori *assumptions as to correct phylogeny of placentals (which represent 'appeals to authority' *sensu *[11]). However, if multiple optimal trees were presented, and the authors explicitly preferred one or a subset of these, we followed this preference [32]. In cases of gene paralogy in molecular analyses, where the same species may be represented in multiple different positions within the same tree, all possible permutations of the positions of each placental taxon were entered.

### Synonymisation

To standardise terminal taxa among source trees, all taxa in all source trees were initially synonymised by hand to species using the taxonomy presented in Wilson and Reeder [7]. In the absence of specific information, subfamilies and families were synonymised with the type species of the genus giving them their names (following [32]). For example, *Bos*, Bovinae and Bovidae were all coded as *Bos taurus*. Terminal taxa that could not be identified to the family-level or below were pruned from the source trees, and source trees with fewer than three taxa remaining were not used. Taxa represented only by common names that did not unequivocally identify families (e.g. 'monkey') were likewise deleted.

Species-level terminal taxa were then synonymised to higher-level terminals using the Perl script synonoTree [32], following Wilson and Reeder's [7] taxonomy. For those source trees where synonymisation resulted in non-monophyletic terminals (i.e. members of the same higher taxon did not form a monophyletic group in the original source tree), synonoTree outputs multiple trees with the non-monophyletic terminal taxa in each of their possible positions.

For the subsidiary analysis, species-level terminals were likewise synonymised to family-level, except that carnivorans and primates were synonymised to order-level, as in LEA. Non-placental terminals were deleted, as were the families Ctenodactylidae, Ctenomyidae, Moschidae, Neobalaenidae and Petromuridae, which were excluded by LEA because their inclusion led to a considerable loss of resolution in their original analysis.

### Establishing Independent Source Trees

Bininda-Emonds et al. [32] advocated that only 'independent evolutionary hypotheses' should be included in a supertree analysis (but see [31] for a critique of Bininda-Emonds et al.'s definition of independence). Source trees that represent the same character and taxon sets (e.g., multiple most-parsimonious trees, or maximum parsimony and maximum likelihood trees of the same dataset) are clearly non-independent. We combined each set of such non-independent source trees into a single 'mini-supertree' [32], for both our full and subsidiary data sets in turn. To identify non-independent source trees (*sensu *[32]), all source trees were initially sorted into groups representing the same character set (e.g. all MTCO1 trees, all 12S + 16S rRNA trees or all DNA-hybridisation trees), with gene names synonymised where possible according to the taxonomy proposed by the Human Genome Organisation Gene Nomenclature Committee [62] and the GeneCards database [63]. We have assumed that different introns, exons or domains of the same gene represent the same non-independent character set in this study, unless there was strong evidence to the contrary. Within each group of non-independent source trees, if multiple trees from the same reference and representing the exact same data set were present (e.g. multiple most parsimonious cladograms), these were combined into a mini-supertree, which could then be used to represent that dataset in the final supertree analysis.

If, after this procedure, any of a group of non-independent source trees or mini-supertrees was a strict taxonomic subset of any other, the taxonomically less inclusive source tree (or trees) was excluded from the final analysis as being redundant. If there was only partial taxonomic overlap between source trees representing the same character set, we did not create a mini-supertree of these, as any lack of resolution in the mini-supertree may be because of insufficient taxonomic overlap, rather than genuine incongruence between the source trees. Instead, these partially overlapping source trees were included separately.

### MRP analyses

We used matrix representation with parsimony (MRP; [22,23]) for both the mini- and overall supertree analyses: each source tree is encoded using additive binary coding, with each taxon coded as '1' if it descends from a particular node in the source tree, '0' if it does not, and '?' if it is not present in that source tree. This procedure is performed for all informative nodes in the source tree. A single matrix containing the combined 'matrix representations' of every source tree is then subjected to parsimony analysis; the resultant most parsimonious tree (or trees) is the supertree, and contains every taxon present in any source tree [22,23]. All MRP matrices were generated using the Perl script SuperMRP.

Within our full dataset, all MRP matrices were produced using 'semi-rooted' MRP coding [37]. This modification of standard MRP coding does not use an all-zero 'MRP outgroup' to root every source tree, but only those where the position of the root is held to have been determined robustly. As such, the method does not enforce questionable rooting decisions present in the source trees, such as rooting based on *a priori *assumptions about the relationships of the in-group. This modification may be particularly advisable for groups where the position of the root remains unclear, such as placentals (see [15]). Here, we consider the presence of non-placental outgroup taxa (such as marsupials and non-mammals) or paralogous genes to represent robust rooting information. We synonymised all such 'real outgroups' to the name 'Real_OG', and used this taxon to root the MRP supertrees. For our subsidiary analysis, we instead followed LEA and used standard MRP coding with the hypothetical, all-zero MRP outgroup common to all source trees to root the supertree [23].

The resultant MRP matrices were analysed using PAUP* 4.0b10 [64]. We used reversible parsimony with all characters weighted equally, unless some of the source trees contained non-monophyletic families, in which case we downweighted the associated MRP characters appropriately. For example, a single non-monophyletic family in two distinct positions in a single initial source tree would be included in two, non-independent source trees (in a different position in each), and the MRP characters corresponding to those trees would each be given a weight of 0.5. Although weighting of MRP characters in proportion to the degree of support for their corresponding nodes has been shown to improve performance [65], we could not implement this in our study due to the non-comparable indices used (e.g. bootstrapping, jackknifing, decay indices, Bayesian posterior probabilities) in different source trees, and the absence of support values of any kind for many source trees.

Branch-and-bound tree searches were used for all our mini-supertree analyses, and the mini-supertree was taken to be the strict consensus of all equally most parsimonious solutions. The final MRP matrices of both full and subsidiary data sets were analysed using the parsimony ratchet [57], with the PAUP* instruction block produced using the Perl script PerlRat. For the full analysis, 20 batches of 500 replicates were carried out, with 25% of the characters randomly chosen to be upweighted by a factor of two in each ratchet replicate, followed by a brute force heuristic search starting from the set of shortest trees found among all 20 batches. The subsidiary matrix was considerably smaller, so 50 batches of 500 replicates were carried out, again followed by a brute force search. TBR branch swapping was employed in all ratchet searches. For the iterative reweighting steps, a maximum of one tree was held at each step, whereas the maximum number of trees for final brute force searches was equal to the product of the number of batches and 1 + the number of replicates.

### Final datasets

The full dataset included 725 trees [see Additional file 3], of which 109 were MRP mini-supertrees, and 54 were due to nonmonophyletic taxa in some source trees. 652 were based on molecular data, 58 on morphology and 15 on combined molecular and morphological data. Following Bininda-Emonds and Sanderson [66], a 'seed tree' was added to ensure sufficient overlap among source trees. This assigned all 115 terminal taxa to their respective orders without specifying any further relationships. Ordinal membership came from Wilson and Reeder [7], except Plesiorycteropodidae (not listed by [7]), which was treated as an additional order, Bibymalagasia, following MacPhee [35]. These tree descriptions were converted into a 'semi-rooted' MRP matrix of 6715 pseudocharacters [see Additional file 4]. We did not differentially weight MRP characters from different source trees, apart from the downweighting of multiple non-independent trees arising because nonmonophyletic families.

The subsidiary data set comprised 466 trees [see Additional file 5], of which 48 were MRP mini-supertrees, and 24 resulted because of nonmonophyletic taxa in some source trees. 408 were based on molecular data, 43 on morphology and ten on combined molecular and morphological data. We again included a 'seed tree', as above. Tree descriptions were converted into a standard MRP matrix of 1857 pseudocharacters [see Additional file 6]. We followed LEA in performing two analyses, one with equal weightings and one in which larger trees were upweighted by a factor of four (this upweighted analysis was the basis for their Figure 1), on the assumption that such trees tend to be of higher quality. In the latter analysis, we upweighted all source trees that were originally upweighted by LEA and that we retained after application of the protocol (53 in total).

The seed trees used in both analyses derive from the taxonomy presented in Wilson and Reeder [7], and therefore violate the source tree collection guidelines (because the taxonomy is not based on an explicit dataset). They were chosen because the taxonomy is a widely used standard for mammals, is fully comprehensive, and has a relatively low information content ensuring that it will be easily overruled by any robust source trees. However, because the taxonomy supports ordinal monophyly, it will bias both analyses slightly in this direction. Nevertheless, as we discuss below and in Results and Discussion, the degree of support for the orders whose monophyly is upheld is much too great to be attributed to the seed trees alone.

### Support values

We calculated the supertree-specific support measure, reduced qualitative support (rQS; [26]) to assess the support for nodes in both the full and subsidiary analyses. This measure is a modified version of qualitative support (QS), as developed by Bininda-Emonds [39], in which support for each supertree clade is calculated by comparing the supertree with each of its source trees in turn. As such, it avoids problems associated with the inherent non-independence of MRP pseudocharacters that renders the use of the more familiar support measures, such as the bootstrap or decay index (DI), invalid [39,67]. Fortunately, QS values are roughly correlated with bootstrap values [39].

For rQS, each supertree clade is supported ('Hard Match') contradicted ('Hard Mismatch'), or is neither supported nor contradicted ('Equivocal) by each source tree. rQS values range from -1 to +1, indicating a greater proportion of hard mismatches and hard matches among the set of source trees, respectively. An rQS value of -1 indicates an unsupported novel clade, the presence of which has been argued by some to be a negative feature of MRP supertrees (e.g. [68]). rQS avoids the problems that affect QS identified by Wilkinson et al. [69]. Other supertree-specific metrics for assessing support, such as V [69], triplet-based methods [70] and modified bootstrap methods [71,72], have also been recently proposed, but are not used here. All rQS values were determined using the Perl script QualiTree [39].

Results from the rQS analyses also confirmed that the inclusion of seed trees had a minimal effect on the topologies of the resultant supertrees. For the full analysis, the seed tree was informative for only 19 of the 113 nodes on the supertree. It directly conflicted with 10 of these 19 nodes, indicating that it was being overruled about half the time, and its removal did not affect rQS values significantly (mean difference between values with and without seed tree = -2.179 × 10^-4^, df = 18, t = -0.641, one-tailed P-value = 0.74). These findings indicate that the supertree is reflecting the signal from the 725 other source trees, rather than the seed tree. Similar results were apparent for the LEA+P analysis, where the seed tree conflicted with five of the 13 informative nodes on this supertree and its removal also did not alter rQS values significantly (mean difference between values with and without seed tree = 7.352 × 10^-5^, df = 12, t = 0.113, one-tailed P-value = 0.46).

For the differentially-weighted subsidiary analysis, we additionally computed DI values for each node, but solely for comparison with the values reported by LEA, given that the measure is not strictly valid in a supertree context. Analyses used the program AutoDecay [73] to specify constraint trees and PerlRat to specify the ratchet search parameters for PAUP*. Because of the large number of nodes to be examined, the ratchet searches were more limited (two runs comprising 20 batches of 100 replicates and one run comprising 5 batches of 200 replicates, for each node) than that used to derive the entire supertree, and the concluding brute force search was omitted. The more limited nature of the searches means that the DI for each node may overestimate the real value in some cases.

We used the normalised partition metric (also known as the Robinson-Foulds topological distance [55,56]) and 'explicitly agree' triplets to quantify the topological differences between: 1) the full supertree (Figure 1), 2) the subsidiary analysis of the LEA references alone, using the 4:1 weighting scheme (Figure 2), 3) the subsidiary analysis, using 1:1 equal weighting (topology not shown), 4) the original LEA combined supertree, 5) the topology of Murphy et al. ([17]; their Figure 1; this is the taxonomically most comprehensive molecular phylogeny of placental mammals currently available), and 6) the combined molecular and morphological topology of Gatesy et al. ([11]; their Figure 4). The normalised partition metric scores were calculated using the perl script partitionMetric, whilst the 'explicitly agree' triplet scores were calculated using COMPONENT [74]; for both metrics, trees pruned to have identical taxon sets for each pairwise comparison.

## Authors' contributions

RMDB collected most of the source trees, carried out all of the analyses and wrote most of the manuscript, as part of an MSc in Advanced Methods in Taxonomy and Biodiversity at Imperial College and the Natural History Museum, London. ORPBE co-supervised RMDB, wrote the Perl scripts, advised on the analyses, and wrote significant portions of the manuscript. MC collected some of the source trees, prepared parts of the supplementary file, and helped write the manuscript. FGRL collected some of the source trees and helped write the manuscript. AP conceived of and developed the research project, supervised RMDB, and wrote significant portions of the manuscript. All authors read and approved the final manuscript.

## Supplementary Material

Additional file 1 RTF file listing all 315 original references used by Liu et al. [29] and reasons for excluding source trees from some of these references, plus an additional 204 references identified as containing valid source trees. Click here for file

Additional file 2 XLS file giving details of all source trees coded from the references listed in Additional file 1. Click here for file

Additional file 3 NEXUS file (can be viewed using a text editor) containing all source trees used in the complete analysis, in parenthetical notation. Weights given to each source tree are also listed. Click here for file

Additional file 4 NEXUS file (can be viewed using a text editor) containing semi-rooted MRP pseudocharacter matrix used in the complete analysis, the weighting scheme used and 50% majority-rule consensus (illustrated in Figure 1) following parsimony analysis of this matrix. Click here for file

Additional file 5 NEXUS file (can be viewed using a text editor) containing all source trees used in the subsidiary LEA analysis, in parenthetical notation. Weights given to each source tree are also listed. Click here for file

Additional file 6 NEXUS file (can be viewed using a text editor) containing standard MRP pseudocharacter matrix used in the complete analysis, 4:1 and 1:1 weighting schemes and 50% majority-rule consensuses (for both weighting schemes; 4:1 consensus illustrated in Figure 2) following parsimony analysis of this matrix. Click here for file
