# Supplementary material for: A higher-level MRP supertree of placental mammals
Source: BMC Evol Biol. 2006 Nov 13;6:93. doi: 10.1186/1471-2148-6-93 (PMC1654192; doi:10.1186/1471-2148-6-93)
Supplement: Additional file 1 — RTF file listing all 315 original references used by Liu et al. [29] and reasons for excluding source trees from some of these references, plus an additional 204 references identified as containing valid source trees. [file 1471-2148-6-93-S1.rtf]

Liu et al. (2001) original reference list

1.	Adkins, R. M. & Honeycutt, R. L. in Primates and Their Relatives in Phylogenetic Pespective (ed MacPhee, R. D. E.) 227-249 (Plenum, New York, 1993).

2.	Aida, Y., Niimi, M., Asahina, M., Okada, K., Nakai, Y. & Ogimoto, K. Identification of a new bovine MHC class II DRB allele by nucleotide sequencing and an analysis of phylogenetic relationships.  Biochem. Biophys. Res. Comm. 209, 981-988 (1995).

3.	Air, G. M., Thompson, E. O., Richardson, B. J. & Sharman, G. B. Amino acid sequences of kangaroo myoglobin and hemoglobin and the date of marsupial-eutherian divergence.  Nature 229, 391-394 (1971).
Not used, as assumes existing tree

4.	Ammerman, L. K. & Hillis, D. M.  A molecular test of bat relationships - monophyly or diphyly.  Syst. Biol. 41, 222-232 (1992).

5.	Arab, S. F., Krohn, K., Lachmund, A., Unsicker, K. & Suter Crazzolara, C.  The gene encoding bovine brain-derived neurotrophic factor (BDNF).  Gene 185, 95-98 (1997).

6.	Arnason, U. & Johnson, E.  The complete mitochondrial DNA sequence of the harbor seal, Phoca vitulina.  J. Mol. Evol. 34, 493-505 (1992).
Not used, as trees are unrooted

7.	Arnason, U. & Ledje, C. in Mammal Phylogeny.  Placentals (eds Szalay, F. S., Novacek, M. J. & McKenna, M. C.) 74-80 (Springer-Verlag, New York, 1993).

8.	Arpagaus, M. et al.  Use of the polymerase chain reaction for homology probing of butyrylcholinesterase from several vertebrates. J. Biol. Chem. 266, 6966-6974 (1991).

9.	Atchley, W. R., Bronner-Fraser, M. & Fitch, W. M.  Molecular evolution of the MyoD family of transcription factors.  Proc. Natl. Acad. Sci. USA 91, 11522-11526 (1994).

10.	Atchley, W. R. & Fitch, W. M.  Myc and max: Molecular evolution of a family of proto-oncogene products and their dimerization partner.  Proc. Natl. Acad. Sci. USA 92, 10217-10221 (1995).

11.	Baker, C. S. & Palumbi, S. R. in Conservation Genetics: Case Histories from Nature (eds Avise, J. C. & Hamrick, J. L.) 11-49 (Chapman and Hall, New York, 1996).

12.	Barnes, L. G. in The Bottlenose Dolphin (eds Leatherwood, S. & Reeves, R.R.) 3-26  (Academic, San Diego 1990.)

13.	Barton, R. A., Purvis, A. & Harvey, P. H.  Evolutionary radiation of visual and olfactory brain systems in primates, bats and insectivores.  Phil. Trans. R. Soc. Lond. B 348, 381-392 (1995).
Not used, as composite of existing trees

14.	Bedford, J. M.  Unusual nature and possible evolutionary implications of the male vesicular gland secretion in the tree shrew, Tupaia glis.  Anat Rec. 247, 199-205 (1997).
Not used, as no explicit tree presented

15.	Beintema, J. J., Schuller, C., Irie, M. & Carsana, A.  Molecular evolution of the ribonuclease superfamily.  Prog. Biophys. Molec. Biol. 51, 165-192 (1988).

16.	Benachenhou-Lahfa, N., Forterre, P. & Labedan, B.  Evolution of glutamate dehydrogenase genes: evidence for two paralogous protein families and unusual branching patterns of the Archaebacteria in the universal tree of life.  J. Mol. Evol. 36, 335-346 (1993).

17.	Bennett, S., Alexander, L. J., Crozier, R. H., & Mackinlay, A. G.  Are megabats flying primates? Contrary evidence from a mitochondrial DNA sequence.  Aust. J. Biol. Sci. 41, 327-332 (1988).

18.	Berrigan, D., Charnov, E. L., Purvis, A. & Harvey, P. H.  Phylogenetic contrasts and the evolution of mammalian life histories.  Evol. Ecol. 7, 270-278 (1993).
Not used, as composite of existing trees

19.	Berti, P. J. & Storer, A. C.  Alignment/phylogeny of the papain superfamily of cysteine proteases.  J. Mol. Biol. 246, 273-283 (1995).

20.	Bosma, P. T. et al.  Multiplicity of glutamic acid decarboxylases (GAD) in vertebrates: molecular phylogeny and evidence for a new GAD paralog.  Mol. Biol. Evol. 16, 397-404 (1999).

21.	Brack, C. M., Duan, W., Hulbert, A. J. & Schreiber, G.  Wallaby transthyretin. Comp. Biochem. Physiol. B 110, 523-529 (1995).

22.	Brauer, K., Schober, W. & Winkelmann, E.  Phylogenetical changes and functional specializations in the dorsal lateral geniculate nucleus (dLGN) of mammals.  J. Hirnforsch 19, 177-87 (1978).
Not used, as no explicitly phylogenetic tree is presented

23.	Bricteux Gregoire, S., Schyns, R. & Florkin, M.  N-terminal amino acid sequence of trypsinogen from the elephant seal Mirounga leonina L. (Carnivora).  Biochim. Biophys. Acta 351, 87-91 (1974).
Not used, as assumes existing tree


24.	Bromham, L., Phillips, M. J. & Penn, D.  Growing up with dinosaurs: molecular dates and the mammalian radiation. TREE 14, 113-118 (1999).
Not used, as assumes existing trees

25.	Bryant, J. D. & McKenna, M. C.  Cranial anatomy and phylogenetic position of Tsaganomys altaicus (Mammalia: Rodentia) from the Hsanda Gol Formation (Oligocene), Mongolia.  Am. Mus. Nov. 3156, 1-42 (1995).

26.	Bubenik, A. B. in Horns, Pronghorns and Antlers: Evolution, Morphology, Physiology and Social Significance (eds Bubenik, A. B & Bubenik, G.A.) 3-113 (Springer-Verlag, New York, 1990).

27.	Bugge, J.  The cephalic arterial system in sciuromorphs with special reference to the systematic classification of rodents.  Acta Anat. Basel 80, 336-361 (1971).
Not used, as assumes existing tree

28.	Bugge, J.  The cephalic arterial system in insectivores, primates, rodents and lagomorphs, with special reference to the systematic classification.  Acta Anat. Suppl. Basel 62, 1-159 (1974).

29.	Bugge, J.  Cephalic arterial pattern in New World edentates and Old World pangolins with special reference to their phylogenetic relationships and taxonomy. Acta Anat. Basel 105, 37-46 (1979).
Not used, as assumes ordinal monophyly a priori

30.	Bulmer, M., Wolfe, K. H. & Sharp, P. M.  Synonymous nucleotide substitution rates in mammalian genes: implications for the molecular clock and the relationship of mammalian orders.  Proc. Natl. Acad. Sci USA 88, 5974-5978 (1991).
Not used, as employs suprafamilial chimeric sequences, therefore assumes a priori monophyly above the family-level

31.	   Butler, P. M. in The Phylogeny and Classification of the Tetrapods, Volume 2.          Mammals (ed. Benton, M. J.) 117-142 (Clarendon, Oxford, 1988).
Not used, as tree is not explicitly phylogenetic

32.	Cao, Y., Adachi, J., Janke, A., Paabo, S. & Hasegawa, M.  Phylogenetic relationships among eutherian orders estimated from inferred sequences of mitochondrial proteins: instability of a tree based on a single gene.  J. Mol. Evol. 39, 519-527 (1994).

33.	Cao, Y. et al.  Conflict among individual mitochondrial proteins in resolving the phylogeny of eutherian orders.  J. Mol. Evol. 47, 307-322 (1998).

34.	Celander, M. & Stegeman, J. J.  Isolation of a cytochrome P450 3A cDNA sequence (CYP3A30) from the marine teleost Fundulus heteroclitus and phylogenetic analyses of CYP3A genes.  Biochem. Biophys. Res. Comm. 236, 306-312 (1997).

35.	Chou, I. T. & Gasser, C. S.  Characterization of the cyclophilin gene family of Arabidopsis thaliana and phylogenetic analysis of known cyclophilin proteins. Plant Mol. Biol. 35, 873-892 (1997).

36.	Claudianos, C. & Campbell, H. D.  The novel Flightless-I gene brings together two gene families, actin-binding proteins related to gelsolin and leucine-rich-repeat proteins involved in ras signal transduction.  Mol. Biol. Evol. 12, 405-414 (1995).

37.	Cooper, A. & Fortey, R.  Reply from A. Cooper and R. Fortey.  TREE 13, 323-324 (1998).
Not used, as it is a composite of existing trees, and assumes ordinal monophyly a priori

38.	Costache, M. et al.  Evolution of fucosyltransferase genes in vertebrates. J. Biol. Chem. 272, 29721-29728 (1997).

39.	Coulier, F. et al. Of worms and men: an evolutionary perspective on the fibroblast growth factor (FGF) and FGF receptor families.  J. Mol. Evol. 44, 43-56 (1997).

40.	Croteau, W., Davey, J. C., Galton, V. A. & St Germain, D. L.  Cloning of the mammalian type II iodothyronine deiodinase.  A selenoprotein differentially expressed and regulated in human and rat brain and other tissues.  J. Clin. Invest. 98, 405-17 (1996).

41.	Cummings, M. P., Otto, S. P. & Wakely, J.  Sampling properties of DNA sequence data in phylogenetic analysis.  Mol. Biol. Evol. 12, 814-822 (1995).
	
42.	Czelusniak, J. et al. Perspectives from amino acid sequences on cladistic relationships among higher taxa of Eutheria.  Curr. Mammal. 2, 545-572 (1990).

43.	Czelusniak, J., Goodman, M., Moncrief, N. D. & Kehoe, S. M.  Maximum parsimony approach to construction of evolutionary trees from aligned homologous sequences.  Meth. Enzymol. 183, 601-615 (1990).

44.	Dawson, S. P., Wilde, C. J., Tighe, P. J. & Mayer, R. J.  Characterization of two novel casein transcripts in rabbit mammary gland.  Biochem. J. 296, 777-784 (1993).

45.	de Jong, W. W.  Molecules remodel the mammalian tree.  TREE 13, 270-275 (1998).
Not used, as asssumes existing trees

46.	de Jong, W. W., Lubsen, N. H. & Kraft, H. J.  Molecular evolution of the eye lens. Progr. Ret. Eye Res. 13, 391-442 (1994).

47.	de Muizon, C.  Are the squalodonts related to the platanistoids?  Proc. San Diego Soc. Nat. Hist. 29, 135-146 (1994).
Not used, as asssumes existing trees

48.	Diaz-Lazcoz, Y. et al.  Evolution of genes, evolution of species: the case of aminoacyl-tRNA synthetases.  Mol. Biol. Evol. 15, 1548-1561 (1998).

49.	Domning, D. P., Ray, C. E. & McKenna, M. C.  Two new Oligocene desmostylians and a discussion of tethytherian systematics.  Smith. Cont. Paleo. 59, 1-56 (1986).
Not used, as assumes ordinal monophyly a priori

50.	Dorner, M. & Paabo, S.  Nucleotide sequence of a marsupial LINE-1 element and the evolution of placental mammals.  Mol. Biol. Evol. 12, 944-948 (1995).

51.	Douzery, E. & Randi, E.  The mitochondrial control region of Cervidae: evolutionary patterns and phylogenetic content.  Mol. Biol. Evol. 14, 1154-1166 (1997).

52.	Dumont, E. R.  Salivary pH and buffering capacity in frugivorous and insectivorous bats.  J. Mammal. 78, 1210-1219 (1997).
Not used, as tree is not explicitly phylogenetic

53.	Duncan, R., Faggart, M. A., Roger, A. J. & Cornell, N. W.  Phylogenetic analysis of the 5-aminolevulinate synthase gene.  Mol. Biol. Evol. 16, 383-396 (1999).

54.	Easteal, S.  The pattern of mammalian evolution and the relative rate of molecular evolution.  Genetics 124, 165-174 (1990).

55.	Erickson, H. P.  Evolution of the tenascin family—implications for function of the C-terminal fibrinogen-like domain.  Persp. Dev. Neurobiol. 2, 9-19 (1994).

56.	Escriva, H. et al.  Rat mammary-gland transferrin: nucleotide sequence, phylogenetic analysis and glycan structure.  Biochem. J. 307, 47-55 (1995).

57.	Feng, D.-F. & Doolittle, R. F.  Converting amino acid alignment scores into measures of evolutionary time: a study of various relationships.  J. Mol. Evol. 44, 361-370 (1997).

58.	Finlay, B. L. & Sengelaub, D. R.  Toward a neuroethology of mammalian vision: ecology and anatomy of rodent visuomotor behavior.  Behav. Brain Res. 3, 133-149 (1981).
Not used, as trees are not explicitly phylogenetic

59.	Fischer, M. S. & Tassy, P. in Mammal Phylogeny.  Placentals (eds Szalay, F. S., Novacek, M. J. & McKenna, M. C.) 217-234 (Springer-Verlag, New York, 1993).
Not used, as ordinal monophyly assumed a priori

60.	Fitch, W. M. & Beintema, J. J.  Correcting parsimonious trees for unseen nucleotide substitutions: the effect of dense branching as exemplified by ribonuclease.  Mol. Biol. Evol. 7, 438-443 (1990).

61.	Flynn, L. J., Jacobs, L. L. & Cheema, I. Q.  Baluchimynae, a new ctenodactyloid rodent subfamily from the Miocene of Baluchistan.  Am. Mus. Nov. 2841, 1-58 (1986).

62.	Flynn, J. J. & Nedbal, M.  Phylogeny of the Carnivora (Mammalia): congruence vs. incompatibility among multiple data sets.  Mol. Phylogenet. Evol. 9, 414-426 (1998).

63.	Fordyce, R. E.  Waipatia maerewhenua, new genus and new species (Waipatiidae, new family), an archaic late Oligocene dolphin (Cetacea: Odontoceti: Platanistoidea) from New Zealand.  Proc. San Diego Soc. Nat. Hist. 29, 147-176 (1994).

64.	Fordyce, R. E. & Barnes, L. G.  The evolutionary history of whales and dolphins.  Annu. Rev. Earth Planet. Sci. 22, 419-455 (1994).
Not used, as assumes existing trees

65.	Friedlander, T. P., Regier, J. C. & Mitter, C.  Phylogenetic information content of five nuclear gene sequences in animals: initial assessment of character sets from concordance and divergence studies.  Syst. Biol. 43, 511-525 (1994).

66.	Fryxell, K. J.  The evolutionary divergence of neurotransmitter receptors and second-messenger pathways.  J. Mol. Evol 41, 85-97 (1995).

67.	Fukami Kobayashi, K., Nosaka, M., Nakazawa, A. & Go, M.  Ancient divergence of long and short isoforms of adenylate kinase: molecular evolution of the nucleoside monophosphate kinase family.  FEBS Lett. 385, 214-220 (1996).

68.	Gamulin, V., Skorokhold, A., Kavson, V., Muller, I. M. & Muller, W. E. G. Experimental indication in favor of the introns-late theory: the receptor tyrosine kinase gene from the sponge Geodia cydonium.  J. Mol. Evol. 44, 242-252 (1997).

69.	Gandolfi, F. et al.  Similarity of an oviduct-specific glycoprotein between different species.  Reprod. Fert. Dev. 5, 433-443 (1993).
Not used, as no explicit tree presented

70.	Garland, Jr., T., Dickerman, A. W., Janis, C. M. & Jones, J. A.  Phylogenetic analysis of covariance by computer simulation.  Syst. Biol. 42, 265-292 (1993).
Not used, as composite of existing trees

71.	Gaskin, D. E.  The Ecology of Whales and Dolphins (Heinemann, London, 1982).
Not used, as tree is not explicitly phylogenetic

72.	Gatesy, J.  More DNA support for a Cetacea/Hippopotamidae clade: the blood-clotting protein gene gamma-fibrinogen.  Mol. Biol. Evol. 14, 537-543 (1997).
 
73.	Gatesy, J., Hayashi, C., Cronin, M. A. & Arctander, P.  Evidence from milk casein genes that cetaceans are close relatives of hippopotamid artiodactyls.  Mol. Biol. Evol. 13, 954-963 (1996).

74.	Gatesy, J., Milinkovitch, M., Waddell, V. & Stanhope, M.  Stability of cladistic relationships between Cetacea and higher-level artiodactyl taxa.  Syst. Biol. 48, 6-20 (1999).

75.	Gaudin, T. J.  The ear region of edentates and the phylogeny of the Tardigrada (Mammalia, Xenarthra).  J. Vert. Paleo. 15, 672-705 (1995).

76.	Gaudin, T. J., Wible, J. R., Hopson, J. A. & Turnbull, W. D.  Reexamination of the morphological evidence for the cohort Epitheria (Mammalia, Eutheria).  J. Mammal. Evol. 3, 31-79 (1996).

77.	Geisler, J. H. & Luo, Z.  The petrosal and inner ear of Herpetocetus sp. (Mammalia: Cetacea) and their implications for the phylogeny and hearing of archaic mysticetes. J. Paleo. 70, 1045-1066 (1996).

78.	Gentry, A. W. & Hooker, J. J. in The Phylogeny and Classification of the Tetrapods, Volume 2.  Mammals (ed Benton, M. J.) 235-272 (Clarendon, Oxford, 1988).

79.	Gershwin, L. J.  The phylogenetic development of anaphylactic activity and homocytotropic antibodies.  Dev. Comp. Immunol. 2, 595-615 (1978).
Not used, as no explicit tree presented

80.	Glanz, W. E. & Anderson, S.  Notes on Bolivian mammals: 7.  A new species of Abrocoma (Rodentia) and relationships of the Abrocomidae.  Am. Mus. Nov. 2991, 1-32 (1990).

81.	Golding, G. B. & Dean, A. M.  The structural basis of molecular adaptation.  Mol. Biol. Evol. 15, 355-369 (1998).

82.	Goodman, M., Romero-Herrera, A. E., Dene, H., Czelusniak, J. & Tashian, R. E. in   Macromolecular Sequences in Systematic and Evolutionary Biology (ed Goodman, M.) 115-191 (Plenum, New York, 1982).

83.	Graur, D.  Towards a molecular resolution of the ordinal phylogeny of the eutherian mammals.  Febs Lett. 325, 152-159 (1993).
Not used, as reference is a review, and trees assume ordinal monophyly a priori

84.	Graur, D., Duret, L. & Gouy, M.  Phylogenetic position of the order Lagomorpha (rabbits, hares and allies).  Nature 379, 333-335 (1996).
Not used, as trees assume ordinal monophyly a priori

85.	Graur, D., Gouy, M. & Duret, L.  Evolutionary affinities of the order Perissodactyla and the phylogenetic status of the superordinal taxa Ungulata and Altungulata.  Mol. Phylogenet. Evol. 7, 195-200 (1997).
Not used, as trees assume ordinal monophyly a priori

86.	Graur, D., Hide, W. A. & Li, W.-H.  Is the guinea pig a rodent?  Nature 351, 649-652 (1991).

87.	Graur, D., Hide, W. A., Zharkikh, A. & Li, W.-H.  The biochemical phylogeny of guinea pigs and gundis, and the paraphyly of the order Rodentia.  Comp. Biochem. Physiol. B 101, 495-498 (1992).
Not used, as same analyses as reference 86 and and reference 147

88.	Graur, D. & Higgins, D. G.  Molecular evidence for the inclusion of cetaceans within the order Artiodactyla.  Mol. Biol. Evol. 11, 357-364 (1994).

89.	Gretarsdottir, S. & Arnason, U.  Evolution of the common cetacean highly repetitive DNA component and the systematic position of Orcaella brevirostris.  J. Mol. Evol. 34, 201-208 (1992).

90.	Gretarsdottir, S. & Arnason, U.  Molecular studies on two variant repeat types of the common cetacean DNA satellite of the sperm whale, and the relationship between Physeteridae (sperm whales) and Ziphiidae (beaked whales).  Mol. Biol. Evol. 10, 306-318 (1993).

91.	Griess, E. A., Rensing, S. A., Grasser, K. D., Maier, U.-G. & Feix, G.  Phylogenetic relationships of HMG box DNA-binding domains.  J. Mol. Evol. 37, 204-210 (1993).

92.	Groves, C. P. & Grubb, P. in Biology and Management of the Cerividae (ed Wemmer, C.) 21-59 (Smithsonian Inst., Washington, 1987).

93.	Hackstein, J. H. P. & van Alen, T. A.  Fecal methanogens and vertebrate evolution. Evolution 50, 559-572 (1996).
Not used, as assumes existing trees

94.	Hafner, M. S.  A biochemical investigation of geomyoid systematics (Mammalia: Rodentia).  Z. Zool. Syst. Evol. 20, 118-130 (1982).

95.	Hartl, G. B. et al.  On the biochemical systematics of selected mammalian taxa: empirical comparison of qualitative and quantitative approaches in the evaluation of protein electrophoretic data.  Z. Zool. Syst. Evol. 28, 191-216 (1990).

96.	Hasegawa, M., Cao, Y., Adachi, J. & Yano, T.  Rodent polyphyly? Nature 355, 595 (1992).
Not used, as assumes ordinal monophyly a priori

97.	Hayzer, D. J.  Immunoglobulin lambda light chain evolution: Igl and Igl-like sequences form three major groups.  Immunogenetics 32, 157-174 (1990).

98.	Hedges, S. B. & Maxson, L. R.  Pancreatic polypeptide and the sister group of birds.  Mol. Biol. Evol. 8, 888-891 (1991).

99.	Hedges, S. B., Moberg, K. D. & Maxson, L. R.  Tetrapod phylogeny inferred from 18S and 28S ribosomal RNA sequences and a review of the evidence for amniote relationships.  Mol. Biol. Evol. 7, 607-633 (1990).

100.	Hewett-Emmett, D, Hopkins, P. J. & Tashian, R. E.  Origins and evolution of the carbonic anhydrase isoenzymes.  Ann. N. Y. Acad. Sci. 429, 338-358 (1984).

101.	Heyning, J. E.  Sperm whale phylogeny revisited: analysis of the morphological evidence.  Mar. Mammal Sci. 13, 596-613 (1997).

102.	Hide, W. A., Chan, L. & Li, W.-H.  Structure and evolution of the lipase superfamily.  J. Lipid Res. 33, 167-178 (1992).

103.	Hollar, L. J. & Springer, M. S.  Old World fruitbat phylogeny: evidence for convergent evolution and an endemic African clade.  Proc. Natl. Acad. Sci. U S A 94, 5716-5721 (1997).

104.	Holmes, E. C.  Different rates of substitution may produce different phylogenies of the eutherian mammals.  J. Mol. Evol. 33, 209-215 (1991).
Not used, as assumes ordinal monophyly a priori

105.	Holmes, R. S. & Massaro, E. J.  Phylogenetic variation of rodent liver esterases.  J. Exp. Zool. 172, 323-334 (1969).

106.	Honeycutt, R. L., Nedbal, M. A., Adkins, R. M. & Janecek, L. L.  Mammalian mitochondrial DNA evolution: a comparison of the cytochrome b and cytochrome c oxidase II genes.  J. Mol. Evol. 40, 260-272 (1995).

107.	Hooker, J. J. in The Evolution of Perissodactyls (eds Prothero, D. R. & Schoch, R. M.) 79-101 (Clarendon, New York, 1989).

108.	Hoss, M. et al.  Molecular phylogeny of the extinct ground sloth Mylodon darwinii. Proc. Natl. Acad. Sci. U S A 93, 181-185 (1996).

109.	Htay, H. H., Sato, K., Tamaki, K., Tsutsumi, H. & Katsumata, Y.  Comparative immunology of mammalian uricase using anti-hog liver uricase.  Comp. Biochem. Physiol. B 92, 583-586 (1989).

110.	Hughes, A. L.  Evolutionary origin and diversification of the mammalian CD1 antigen genes.  Mol. Biol. Evol. 8, 185-201 (1991).

111.	Hughes, A. L.  Evolution of the interleukin-1 gene family in mammals.  J. Mol. Evol. 39, 6-12 (1994).

112.	Hughes, A. L.  The evolution of the type I interferon gene family in mammals.  J. Mol. Evol. 41, 539-548 (1995).

113.	Hughes, A. L.  Gene duplication and recombination in the evolution of mammalian Fc receptors.  J. Mol. Evol. 43, 4-10 (1996).

114.	Hughes, A. L., Hughes, M. K., Howell, C. Y. & Nei, M.  Natural selection at the class II major histocompatibility complex loci of mammals.  Phil. Trans. R. Soc. Lond. B 346, 359-366 (1994).

115.	Hughes, A. L. & Yeager, M.  Coordinated amino acid changes in the evolution of mammalian defensins.  J. Mol. Evol. 44, 675-682 (1997).

116.	Hutcheon, J. M., Kirsch, J. A. W. & Pettigrew, J. D.  Base compositional biases and the bat problem.  III. The question of microchiropteran monophyly.  Phil. Trans. R. Soc. B. 353, 607-617 (1998).  

117.	Iwasaki, H., Suzuki, Y. & Sinohara, H.  Cloning and sequencing of cDNAs encoding plasma alpha-macroglobulin and murinoglobulin from guinea pig: implications for molecular evolution of alpha-macroglobulin family.  J. Biochem. 120, 1167-1175 (1996).

118.	Jaeger, J.-J. in The Phylogeny and Classification of the Tetrapods, Volume 2.  Mammals (ed Benton, M. J.) 177-199 (Clarendon, Oxford, 1988).

119.	Janis, C. M.  New ideas in ungulate phylogeny and evolution.  TREE 3, 291-297 (1988).
Not used, as review article

120.	Janis, C. M. & Scott, K. M.  The interrelationships of higher ruminant families with special emphasis on the members of the Cervoidea.  Am. Mus. Nov. 2893, 1-86 (1987).

121.	Janke, A. & Arnason, U.  The complete mitochondrial genome of Alligator mississippiensis and the separation between recent Archosauria (birds and crocodiles).  Mol. Biol. Evol. 14, 1266-1272 (1997).

122.	Johnstone, E. M., Chaney, M. O., Norris, F. H., Pascual, R., & Little, S. P. Conservation of the sequence of the Alzheimer's disease amyloid peptide in dog, polar bear and five other mammals by cross-species polymerase chain reaction analysis.  Mol. Brain Res. 10, 299-305 (1991).

123.	Kagan, R. M., McFadden, H. J., McFadden, P. N., O'Connor, C., & Clarke, S. Molecular phylogenetics of a protein repair methyltransferase.  Comp. Biochem. Physiol. B 117, 379-385 (1997).

124.	Kass, D. H., Kim, J. & Deininger, P. L.  Sporadic amplification of ID elements in rodents.  J. Mol. Evol. 42, 7-14 (1996).
Not used, as reference assumes an existing tree

125.	Kass, D. H., Kim, J., Rao, A. & Deininger, P. L.  Evolution of B2 repeats: the muroid explosion.  Genetica 99, 1-13 (1997).
Not used, as reference assumes an existing tree

126.	Kasuya, T.  Systematic consideration of recent toothed whales based on the morphology of tympano-periotic bone.  Sci. Rep. Whales Res. Inst. Tokyo 25, 1-103 (1973).
Not used, as trees presented are not explicitly phylogenetic

127.	Katz, L. A.  Transkindom transfer of the phosphoglucose isomerase gene.  J. Mol. Evol. 43, 453-459 (1996).

128.	Kawamoto, T. et al.  Structural and phylogenetic analyses of RGD-CAP/beta Ig-h3, a fasciclin-like adhesion protein expressed in chick chondrocytes.  Biochim. Biophys. Acta Gene Struct. Express. 1395, 288-292 (1998).

129.	Kay, R. F., Thewissen, J. G. M. & Yoder, A. D.  Cranial anatomy of Ignacius graybullianus and the affinities of the Plesiadapiformes.  Am. J. Phys. Anthropol. 89, 477-498 (1992).

130.	Kehoe, J. M. & Capra, J. D.  Phylogenetic aspects of immunoglobulin variable region diversity.  Contemp. Top. Mol. Immunol. 3, 143-159 (1974).
Not used, as trees presented are not explicitly phylogenetic

131.	Kirsch, J. A. W., Flannery, T. F., Springer, M. S. & Lapointe, F. J.  Phylogeny of the Pteropodidae (Mammalia, Chiroptera) based on DNA hybridization, with evidence for bat monophyly.  Aust. J. Zool. 43, 395-428 (1995).

132.	Koh, H. S.  Morphometric analyses on 24 species (13 families of six orders) of Korean mammals.  Korean J. Zool. 32, 14-21 (1989).
Not used, as trees presented are not explicitly phylogenetic

133.	Koide, Y., Papkoff, H. & Kawauchi, H.  Complete amino acid sequences of follitropin and lutropin in the ostrich, Struthio camelus.  Eur. J. Biochem. 240, 262-267 (1996).

134.	Korth, W. W.  The Tertiary Record of Rodents in North America  (Plenum, New York, 1994).
Not used, as trees presented are not explicitly phylogenetic

135.	Kraus, F. & Miyamoto, M. M.  Rapid cladogenesis among the pecoran ruminants: evidence from mitochondrial DNA sequences.  Syst. Zool. 40, 117-130 (1991).

136.	Krawczak, M., Wacey, A. & Cooper, D. N.  Molecular reconstruction and homology modeling of the catalytic domain of the common ancestor of the haemostatic vitamin-K-dependent serine proteinases.  Hum. Genet. 98, 351-370 (1996).
Not used, as assumes an existing tree

137.	Kuma, K. & Miyata, T.  Mammalian phylogeny inferred from multiple protein data. Jpn. J. Genet. 69, 555-566 (1994).

138.	Kutal, M.  Structure of the palatine tonsil in European insectivores.  Adv. Otorhinolaryngol. 47, 59-63 (1992).
Not used, as assumes ordinal monophyly a priori

139.	Lamb, J. G., Straub, P. & Tukey, R. H.  Cloning and characterization of cDNAs encoding mouse Ugt1.6 and rabbit Ugt1.6: differential induction by 2,3,7,8-tetrachlorodibenzo-p-dioxin.  Biochemistry 33, 10513-10520 (1994).

140.	Landan, G., Bdolah, A., Wollberg, Z., Kochva, E. & Graur, D.  Evolution of the sarafotoxin-endothelin superfamily of proteins.  Toxicon 29, 237-244 (1991).

141.	Lapointe, F.-J., Kirsch, J. A. W. & Hutcheon, J. M.  Total evidence, consensus, and bat phylogeny: a distance-based approach.  Mol. Phylogenet. Evol. 11, 55-66 (1999).

142.	Lara, M. C., Patton, J. L. & daSilva, M. N. F.  The simultaneous diversification of South American echimyid rodents (Hystricognathi) based on complete cytochrome b sequences.  Mol. Phylogenet. Evol. 5, 403-413 (1996).

143.	Lavergne, A., Douzery, E., Stichler, T., Catzeflis, F. M. & Springer, M. S. Interordinal mammalian relationships: evidence for paenungulate monophyly is provided by complete mitochondrial 12S rRNA sequences.  Mol. Phylogenet. Evol. 6, 245-258 (1996).

144.	Leinders, J.  Hoplitomerycidae fam. Nov. (Ruminantia, Mammalia) from Neogene fissure fillings in Gargano (Italy).  Part 1: The cranial osteology of Hoplitomeryx gen. Nov. and a discussion on the classification of pecoran families.  Scripta Geol. 70, 1-68 (1983).

145.	Lester, K. S., Hand, S. J. & Vincent, F.  Adult phyllostomid (bat) enamel by scanning electron microscopy: with a note on dermopteran enamel.  Scan. Microscopy 2, 371-384 (1988).
Not used, as no explicit tree presented

146.	Li, M. D. & Ford, J. J.  A comprehensive evolutionary analysis based on nucleotide and amino acid sequences of the alpha- and beta-subunits of glycoprotein hormone gene family.  J. Endocrinol. 156, 529-542 (1998).

147.	Li, W.-H., Hide, W. A., Zharkikh, A., Ma, D. P. & Graur, D.  The molecular taxonomy and evolution of the guinea pig.  J. Hered. 83, 174-181 (1992).

148.	Lioupis, A., Wallis, O. C. & Wallis, M.  Cloning and characterisation of the gene encoding red deer (Cervus elaphus) growth hormone: implications for the molecular evolution of growth hormone in artiodactyls.  J. Mol. Endocrinol. 19, 259-266 (1997).
	Not used, as reference assumes an existing tree

149.	Lipscomb, D. L., Farris, J. S., Kallersjo, M. & Tehler, A.  Support, ribosomal sequences and the phylogeny of the eukaryotes.  Cladistics 14, 303-338 (1998).

150.	Liu, F.-G. R. & Miyamoto, M. M.  Phylogenetic assessment of molecular and morphological data for eutherian mammals.  Syst. Biol. 48, 54-64 (1999).

151.	Lowenstein, J. M., Sarich, V. M. & Richardson, B. J.  Albumin systematics of the extinct mammoth and Tasmanian wolf.  Nature 291, 409-411 (1981).

152.	Luckett, W. P. in Proceedings, Fifth International Bat Conference (eds Wilson, D. E. & Gardener, A. L.) 245-265 (Texas Tech, Lubbock, 1980).

153.	Luckett, W. P. & Hartenberger, J. L.  Monophyly or polyphyly of the order Rodentia: possible conflict between morphological and molecular interpretations.  J. Mammal. Evol. 1, 127-147 (1993).
	Not used, as ordinal monophyly a priori

154.	Luo, Z. X. & Marsh, K.  Petrosal (periotic) and inner ear of a Pliocene kogiine whale (Kogiinae, Odontoceti): implications on relationships and hearing evolution of toothed whales.  J. Vert. Paleo. 16, 328-348 (1996).

155.	Lynch, M.  Mutation and accumulation in nuclear, organelle, and prokaryotic transfer RNA genes.  Mol. Biol. Evol. 14, 914-925 (1997).
Not used, as assumes existing tree topology

156.	Macey, J. R., Larson, A., Ananjeva, N. B., Rang, Z. & Papenfuss, T. J.  Two novel gene orders and the role of light-strand replication in rearrangement of the vertebrate mitochondrial genome.  Mol. Biol. Evol. 14, 91-104 (1997).
Not used, as assumes existing tree topology

157.	MacPhee, R. D. E.  Morphology, adaptations, and relationships of Plesiorycteropus, and a diagnosis of a new order of eutherian mammals.  Bull. Am. Mus. Nat. Hist. 220, 1-214 (1994).

158.	Madsen, O., Deen, P. M. T., Pesole, G., Saccone, C. & de Jong, W. W.  Molecular evolution of mammalian aquaporin-2: further evidence that elephant shrew and aardvark join the paenungulate clade.  Mol. Biol. Evol 14, 363-371 (1997).

159.	Marshall, C. J.  Evolutionary relationships among the serpins.  Phil. Trans. R. Soc. Lond. B 342, 101-119 (1993).

160.	Martignetti, J. A. & Brosius, J.  Neural BC1 RNA as an evolutionary marker: guinea pig remains a rodent.  Proc. Natl. Acad. Sci. U S A 90, 9698-9702 (1993).
Not used, as reference assumes an existing tree

161.	Matthee, C. A. & Ribinson, T. J.  Molecular phylogeny of the springhare, Pedetes capensis, based on mitochondrial DNA sequences.  Mol. Biol. Evol. 14, 20-29 (1997).

162.	McKenna, M. C. in Phylogeny of the Primates (eds Luckett, W. P. & Szalay, F. S.) 21-46 (Plenum, New York, 1975). 
	Not used, as reference assumes ordinal monophyly a priori

163.	McKenna, M. C. in Molecules and Morphology in Evolution: Conflict or Compromise? (ed Patterson, C.) 55-93 (Cambridge Univ., Cambridge, 1987).
Not used, as all cladograms present were produced by hand

164.	McKenna, M. C.  The alpha crystallin A chain of eye lens and mammalian phylogeny.  Annal. Zool. Fenn. 28, 349-360 (1991).
Not used, as all cladograms present were produced by hand

165.	McLeod, S. A., Whitmore, Jr., F. C. & Barnes, L. G. in The Bowhead Whale (eds. Burns, J. J., Montague, J. J. & Cowles, C. J.) 45-70 (Society for Marine Mammalogy., Lawrence, 1993).  

166.	Meng, J.  The auditory region of Reithroparamys delicatissimus (Mammalia, Rodentia) and its systematic implications.  Am. Mus. Nov. 2972, 1-35 (1990).

167.	Meng, J., Wyss, A. R., Dawson, M. R. & Zhai, R.  Primitive fossil rodent from Inner Mongolia and its implications for mammalian phylogeny.  Nature 370, 134-136 (1994).
Not used, as monophyly of extant orders assumed a priori

168.	Messenger, S. L. & McGuire, J. A.  Morphology, molecules, and the phylogenetics of cetaceans.  Syst. Biol. 47, 90-124 (1998).

169.	Messer, M., Weiss, A. S., Shew, D. C. & Westerman, M.  Evolution of the monotremes: phylogenetic relationship to marsupials and eutherians, and estimation of divergence dates based on -lactalbumin amino acid sequences.  J. Mammal. Evol. 5, 95-105 (1998).

170.	Milinkovitch, M. C.  DNA-DNA hybridizations support ungulate ancestry of Cetacea.  J. Evol. Biol. 5, 149-160 (1992).
Not used, as no explicit tree presented

171.	Milinkovitch, M. C.  Molecular phylogeny of cetaceans prompts revision of morphological transformations.  TREE 10, 328-334 (1995).
Not used, as reference assumes existing trees

172.	Milinkovitch, M. C., Meyer, A. & Powell, J. R.  Phylogeny of all major groups of cetaceans based on DNA sequences from three mitochondrial genes.  Mol. Biol. Evol. 11, 939-948 (1994).

173.	Milinkovitch, M. C. & Thewissen, J. G. M.  Evolutionary biology - Even-toed fingerprints on whale ancestry.  Nature 388, 622-624 (1997).
Not used, as reference is a review article

174.	Miyamoto, M. M.  A congruence study of molecular and morphological data for eutherian mammals.  Mol. Phylogenet. Evol. 6, 373-390 (1996).
Not used, as all trees presented in reference are unrooted

175.	Miyamoto, M. M. & Goodman, M.  Biomolecular systematics of eutherian mammals: phylogenetic patterns and classification.  Syst. Zool. 35, 230-240 (1986).

176.	Modi, W. S., Gallagher, D. S. & Womack, J. E.  Evolutionary histories of highly repeated DNA families among the Artiodactyla (Mammalia).  J. Mol. Evol. 42, 337-349 (1996).
Not used, as reference assumes existing tree

177.	Montgelard, C., Catzeflis, F. M. & Douzery, E.  Phylogenetic relationships of artiodactyls and cetaceans as deduced from the comparison of cytochrome b and 12S rRNA mitochondrial sequences.  Mol. Biol. Evol. 14, 550-559 (1997).

178.	Montegelard, C., Ducrocq, S. & Douzery, E.  What is a suiform (Artiodactyla)? Mol. Phylogenet. Evol. 9, 528-532 (1998).

179.	Muizon, C. de.  A new Ziphiidae (Cetacea) from the Early Miocene of Washington State (USA) and phylogenetic analysis of the major groups of odontocetes.  Bull. Mus. Natn. Hist. Nat. Paris 12, 279-326 (1991).

180.	Nakatani, T., Suzuki, Y., Yoshida, K. & Sinohara, H.  Molecular cloning and sequence analysis of cDNA encoding plasma alpha-1-antiproteinase from Syrian hamster: implications for the evolution of Rodentia.  Biochim. Biophys. Acta 1263, 245-248 (1995).

181.	Nakayama, S. & Kresinger, R. H.  Evolution of EF-hand calcium-modulated proteins based on protein sequences: calmodulin dendrograms show significant lack of parallelism.  J. Mol. Evol. 36, 458-476 (1993).

182.	Naylor, G. J. P. & Brown, W. M.  Amphioxus mitochondrial DNA, chordate phylogeny, and the limits of inference based on comparison of sequences.  Syst. Biol. 47, 61-76 (1998).

183.	Nedbal, M. A., Honeycutt, R. L. & Schlitter, D. A.  Higher-level systematics of rodents (Mammalia, Rodentia): evidence from the mitochondrial 12S rRNA gene.  J. Mammal. Evol. 3, 201-237 (1996).

184.	Nelson, D. R. & Strobel, H. W.  Evolution of cytochrome P-450 proteins.  Mol. Biol. Evol. 4, 572-593 (1987).

185.	Newton, G. et al.  Characterization of human and mouse cartilage oligomeric matrix protein.  Genomics 24, 435-439 (1994).

186.	Novacek, M. J. in Proceedings, Fifth International Bat Research Conference (eds Wilson, D. E. & Gardener, A. L.) 317-330 (Texas Tech, Lubbock, 1980).

187.	Novacek, M. J.  Aspects of the morphology of the cochlea in microchiropteran bats: an investigation of character transformation.  Bull. Am. Mus. Nat. Hist. 206, 84-100.
Not used, as reference assumes an existing tree

188.	Novacek, M. J.  Mammalian phylogeny: shaking the tree.  Nature 356, 121-125 (1992).
Not used, as reference is a review of existing data

189.	Novacek, M. J. in Interpreting the Hierarchy of Nature (eds Grande, L. & Rieppel, O.) 85-131 (Academic, New York, 1994).
Not used, as reference assumes ordinal monophyly a priori

190.	Ohnishi, K.  A tentative evolutionary tree of mammalian orders constructed by Hennigian comparison of the amino acid sequences of alpha-crystallin A chain, myoglobin, and hemoglobin alpha chain.  Sci. Rep. Niigata Univ. D 28, 19-32 (1991).

191.	O'hUigin, C., Sultmann, H., Tichy, H. & Murray, B. W.  Isolation of Mhc Class II DMA and DMB cDNA sequences in a marsupial: the gray short-tailed opossum (Monodelphis domestica).  J. Mol. Evol. 47, 578-585 (1998).

192.	Oka, K. et al.  Mouse very-low-density-lipoprotein receptor (VLDLR) cDNA cloning, tissue-specific expression and evolutionary relationship with the low-density-lipoprotein receptor.  Eur. J. Biochem. 224, 975-982 (1994).

193.	Omoe, K. & Endo, A.  Relationship between the monosomy X phenotype and Y-linked ribosomal protein S4 (Rps4) in several species of mammals: a molecular evolutionary analysis of Rps4 homologs.  Genomics 31, 44-50 (1996).

194.	Ono, H., Figueroa, F., O'hUigin, C. & Klein, J.  Cloning of the beta-microglobulin gene in the zebrafish.  Immunogenetics 38, 1-10 (1993).

195.	Palley, L. S., Schlossman, S. F. & Letvin, N. L.  Common tree shrews and primates share leukocyte membrane antigens.  J. Med. Primatol. 13, 67-71 (1984).
Not used, as no explicit tree is presented in the reference

196.	Pan, F. M., Chang, W. C., Lu, S. F., Hsu, A. L. & Chiou, S. H.  Sequence analysis of one major basic beta-crystallin (beta-B-p) of amphibian lenses: evolutionary comparison and phylogenetic relatedness between beta- and gamma-crystallins. Biochem. Biophys. Res. Comm. 217, 940-949 (1995).

197.	Patterson, B., Segall, W., Turnbull, W. D. & Gaudin, T. J.  The ear region in xenarthrans (equals Edentata: Mammalia): Part II.  Pilosa (sloths, anteaters), palaeanodonts, and a miscellany.  Fieldiana Geol. 24, 1-79 (1992).
Not used, as trees presented are not explicitly phylogenetic

198.	Patton, S. J., Luke, G. N. & Holland, P. W. H.  Complex history of a chromosomal paralogy region: insights from Amphioxus aromatic amino acid hydroxylase genes and insulin-related genes.  Mol. Biol. Evol. 15, 1373-1380 (1998).

199.	Penny, D., Hendy, M. D. & Steel, M. A. in Phylogenetic Analysis of DNA Sequences (eds Miyamoto, M. M. & Cracraft, J.) 153-183 (Oxford, New York, 1991).

200.	Perrin, W. F.  Development and homologies of head stripes in the delphinoid cetaceans.  Mar. Mammal Sci. 13, 1-43 (1997).

201.	Pesole, G., Sbisa, E., Mignotte, F. & Saccone, C.  The branching order of mammals: phylogenetic trees inferred from nuclear and mitochondrial molecular data.  J. Mol. Evol. 33, 537-542 (1991).

202.	Pettigrew, J. D.  Flying lemurs and other animals.  Nature 346, 520 (1990).
Not used, as reference does not present an explicit tree, and ordinal monophyly is assumed a priori

203.	Pettigrew, J. D.  Wings or brain?  Convergent evolution in the origins of bats.  Syst. Zool. 40, 199-216 (1991).

204.	Piccinini, M. et al.  Primary structure and oxygen-binding properties of the hemoglobin from the lesser hedgehog tenrec (Echinops telfairi, Zalambdodonta): evidence for phylogenetic isolation.  Biol. Chem. Hoppe-Seyler 372, 975-990 (1991).

205.	Pierson, E. D.  Molecular Systematics of the Microchiroptera: Higher Taxon Relationships and Biogeography (Ph.D. Diss., Univ. California, Berkeley, 1986).

206.	Pilleri, G., Gihr, M. & Kraus, C.  The organ of hearing in Cetacea: II. Paleobiological evolution.  Invest. Cetacea 22, 5-185 (1989).
Not used, as trees presented are not explicitly phylogenetic

207.	Piotte, C. P., Marshall, C. J., Hubbard, M. J., Collet, C. & Grigor, M. R.  Lysozyme and alpha-lactalbumin from the milk of a marsupial, the common brush-tailed possum (Trichosurus vulpecula).  Biochim. Biophys. Acta 1336, 235-242 (1997).

208.	Poulin, R.  Phylogeny, ecology, and the richness of parasite communities in vertebrates.  Ecol. Mono. 65, 283-302 (1995).
Not used, as tree is a composite of existing phylogenies

209.	Pouliot, Y.  Phylogenetic analysis of the cadherin superfamily.  Bioessays 14, 743-748 (1992).

210.	Prothero, D. R. in Mammal Phylogeny.  Placentals (eds Szalay, F. S., Novacek, M. J. & McKenna, M. C.) 173-181 (Springer-Verlag, New York, 1993).
Not used, as reference is a review of previous studies

211.	Pumo, D. E. et al.  Complete mitochondrial genome of a neotropical fruit bat, Artibeus jamaicensis, and a new hypothesis of the relationships of bats to other eutherian mammals.  J. Mol. Evol. 47, 709-717 (1998).

212.	Purvis, A. & Bromham, L.  Estimating the transition/transversion ratio from independent pairwise comparisons with an assumed phylogeny.  J. Mol. Evol. 44, 112-119 (1997).
Not used, as reference assumes an existing tree

213.	Purvis, A. & Harvey, P. H.  Mammal life-history evolution: a comparative test of Charnov's model.  J. Zool. 237, 259-283 (1995).
Not used, as tree is a composite of existing phylogenies

214.	Queralt, R. et al.  Evolution of protamine P1 genes in mammals.  J. Mol. Evol. 40, 601-607 (1995).

215.	Reiss, K. Z.  Myology of the feeding apparatus of myrmecophagid anteaters (Xenarthra: Myrmecophagidae).  J. Mammal. Evol. 4, 87-117 (1997).
Not used, as reference assumes an existing tree

216.	Reizer, J., Reizer, A. & Saier, M. H., Jr.  A functional superfamily of sodium/solute symporters.  Biochim. Biophys. Acta 1197, 133-166 (1994).

217.	Reynolds, P. S.  Phylogenetic analysis of surface areas of mammals.  J. Mammal. 78, 859-868 (1997).
Not used, as tree is a composite of existing phylogenies

218.	Rodriguez-Garcia, M. I., Kozak, C. A., Morgan, R. O. & Fernandez, M. P.  Mouse annexin V chromosomal localization, cDNA sequence conservation, and molecular evolution.  Genomics 31, 151-157 (1996).

219.	Roldan, E. R. S. & Gomendio, M.  The Y chromosome as a battle ground for sexual selection.  TREE 14, 58-62 (1998).
Not used, as reference assumes an existing tree

220.	Rolfsmeier, M., Haseltine, C., Bini, E., Clark, A. & Blum, P.  Molecular characterization of the alpha-glucosidase gene (malA) from the hyperthermophilic archaeon Sulfolobus solfataricus.  J. Bacteriol. 180, 1287-1295 (1998).

221.	Romero Herrera, A. E., Lehmann, H., Joysey, K. A. & Friday, A. E.  Molecular evolution of myoglobin and the fossil record: a phylogenetic synthesis.  Nature 246, 389-395 (1973).

222.	Rubio, N., Sharp, P. M., Rits, M., Zahedi, K. & Whitehead, A. S.  Structure, expression, and evolution of guinea-pig serum amyloid-P component and C-reactive protein.  J. Biochem. 113, 277-284 (1993).

223.	Rypniewski, W. R., Perrakis, A., Vorgias, C. E. & Wilson, K. S.  Evolutionary divergence and conservation of trypsin.  Prot. Engineer. 7, 57-64 (1994).

224.	Sabeur, G., Macaya, G., Kadi, F. & Bernardi, G.  The isochore patterns of mammalian genomes and their phylogenetic implications.  J. Mol. Evol. 37, 93-108 (1993).
Not used, as reference assumes existing trees

225.	Sakai, T.  The mammalian Harderian gland: morphology, biochemistry, function and phylogeny.  Arch. Histol. Jpn. 44, 299-333 (1981).
Not used, as reference does not present an explicit tree

226.	Sarich, V. M.  Generation time and albumin evolution.  Biochem. Genet. 7, 205-212 (1972).
Not used, as reference assumes an existing tree

227.	Sarich, V. M. in Evolutionary Relationships Among Rodents (eds Luckett, W. P. & Hartenberger, J.-L.) 423-452 (Plenum, New York, 1984).

228.	Sayama, K. et al.  Phylogenetic relationships among laboratory animals deduced from basement membrane type IV collagen antigens.  Zool. Sci. 8, 359-370 (1991).

229.	Schmidt, T. R., Jaradat, S. A., Goodman, M., Lomax, M. I. & Grossman, L. I. Molecular evolution of cytochrome c oxidase: rate variation among subunit VIa isoforms.  Mol. Biol. Evol. 14, 595-601 (1997).

230.	Schreiber, A., Erker, D. & Bauer, K.  Artiodactylan phylogeny: an immunogenetic study based on comparative determinant analysis.  Exp. Clin. Immunogenet. 7, 234-243 (1990).

231.	Schreiber, A., Erker, D. & Bauer, K.  Eutherian phylogeny from a primate perspective.  Biol. J. Linn. Soc. 51, 359-376 (1994).

232.	Schrenzel, M. D., King, D. P., McKnight, M. L. & Ferrick, D. A.  Characterization of horse (Equus caballus) immunoglobulin mu chain-encoding genes. Immunogenetics 45, 386-393 (1997).

233.	Seery, L. T., Nestor, P. V. & Fitzgerald, G. A.  Molecular evolution of the aldo-keto reductase gene superfamily.  J. Mol. Evol 46, 139-146 (1998).

234.	Segade, F., Hurle, B., Claudio, E., Ramos, S. & Lazo, P. S.  Identification of an additional member of the cytochrome c oxidase subunit VIIa family of proteins.  J. Biol. Chem. 271, 12343-12349 (1996).

235.	Serdobova, I. M. & Kramerov, D. A.  Using short retroposons as phylogenetic markers.  Doklady Akademii Nauk 335, 664-667 (1994).
Not used, as reference assumes an existing tree

236.	Shafqat, J. et al.  Pea formaldehyde-active class III alcohol dehydrogenase: common derivation of the plant and animal forms but not of the corresponding ethanol-active forms (class I and P).  Proc. Natl. Acad. Sci. USA 93, 5595-5599 (1996).

237.	Shimamura, M. et al.  Molecular evidence from retroposons that whales form a clade within even-toed ungulates.  Nature 388, 666-670 (1997).

238.	Shimmin, L. C., Mai, P. & Li, W.-H.  Sequences and evolution of human and squirrel monkey blue opsin genes.  J. Mol. Evol. 44, 378-382 (1997).
 
239.	Shimura, E. & Numachi, K. I.  Genetic variability and differentiation in the toothed whales.  Sci. Rep. Whales Res. Inst. Tokyo 38, 141-164 (1987).

240.	Shiu, S. Y. W., Ng, N. & Pang, S. F.  A molecular perspective of the genetic relationships of G-protein coupled melatonin receptor subtypes.  J. Pineal Res. 20, 198-204 (1996).

241.	Shoshani, J.  Mammalian phylogeny: comparison of morphological and molecular results.  Mol. Biol. Evol. 3, 222-242 (1986).
Not used, as reference assumes ordinal monophyly a priori

242.	Shoshani, J. in Ongules/Ungulates 91 (ed Spitz, F. et al.) 103-112 (Soc. Franc. Etude Protec. Mamm., Paris, 1992).
Not used, as reference assumes ordinal monophyly a priori

243.	Shoshani, J. in Mammal Phylogeny.  Placentals (eds Szalay, F. S., Novacek, M. J. & McKenna, M. C.) 235-256 (Springer-Verlag, New York, 1993).
Not used, as reference assumes ordinal monophyly a priori

244.	Shoshani, J., Groves, C. P., Simons, E. L. & Gunnell, G. F.  Primate phylogeny: morphological vs. molecular results.  Mol. Phylogenet. Evol. 5, 102-154 (1996).

245.	Shoshani, J. & McKenna, M. C.  Higher taxonomic relationships among extant mammals based on morphology, with selected comparisons of results from molecular data.  Mol. Phylogenet. Evol. 9, 572-584 (1998).

246.	Simmons, N. B.  Morphology, function, and phylogenetic significance of pubic nipples in bats (Mammalia: Chiroptera).  Am. Mus. Nov. 3077, 1-33 (1993).
Not used, as reference assumes existing trees

247.	Simmons, N. B.  The case of chiropteran monophyly.  Am. Mus. Nov. 3103, 1-54 (1994).
Not used, as reference is a review of previous studies

248.	Simmons, N. B.  A reappraisal of interfamilial relationships of bats.  In Bat Biology and Conservation (ed Kunz, T. H. & Racey, P. A.), 3-26 (Smithsonian Inst., Washington, 1998).

249.	Simmons, N. B. & Geisler, J. H.  Phylogenetic relationships of Icaronycteris, Archaeonycteris, Hassianycteris, and Palaeochiropteryx to extant bat lineages, with comments on the evolution of echolocation and foraging strategies in Microchiroptera.  Bull. Am. Mus. Nat. Hist. 235, 4-182 (1998).

250.	Sitnikova, T. & Nei, M.  Evolution of immunoglobulin kappa chain variable region genes in vertebrates.  Mol. Biol. Evol. 15, 50-60 (1998).

251.	Sitnikova, T. & Su, C.  Coevolution of immunoglobin heavy- and light-chain variable-region gene families.  Mol. Biol. Evol. 15, 617-625 (1998). 

252.	Slade, R. W., Hale, P. T., Francis, D. I., Graves, J. A. M. & Sturm, R. A.  The marsupial MHC: the tammar wallaby, Macropus eugenii, contains an expressed DNA-like gene on chromosome 1.  J. Mol. Evol. 38, 496-505 (1994).

253.	Slade, R. W., Moritz, C. & Heideman, A.  Multiple nuclear-gene phylogenies: application to pinnipeds and comparison with a mitochondrial DNA gene phylogeny.  Mol. Biol. Evol. 11, 341-356 (1994).

254.	Slipjer, E. J.  Die Cetaceen.  Vergleichend-Anatomisch und Systematisch (Asher, Amsterdam, 1973).
Not used, as trees presented are not explicitly phylogenetic

255.	Smith, J. D.  Chiropteran evolution.  Spec. Publ. Mus. Texas Tech Univ. 10, 49-69 (1976).
Not used, as trees presented are not explicitly phylogenetic

256.	Smith, K. K.  Comparative patterns of craniofacial development in eutherian and metatherian mammals.  Evolution 51, 1663-1678 (1997).
Not used, as no explicit tree is presented

257.	Snyder, M. J. & Maddison, D. R.  Molecular phylogeny of glutathione-S-transferases.  DNA Cell Biol. 16, 1373-1384 (1997).

258.	Somers, G. R. et al.  Chromosomal localization of the human P2Y-6 purinoceptor gene and phylogenetic analysis of the P2y purinoceptor family.  Genomics 44, 127-130 (1997).

259.	Sparrow, D. A. et al.  Plasma lipid transport in the hedgehog: partial characterization of structure and function of apolipoprotein A-I.  J. Lipid Res. 36, 485-495 (1995).

260.	Spotila, L. D. et al.  Sequence analysis of the ZFY and sox genes in the turtle, Chelydra serpentina.  Mol. Phylogenet. Evol. 3, 1-9 (1994).

261.	Springer, M. S., Amrine, H. M., Burk, A. & Stanhope, M. J.  Additional support for Afrotheria and Paenungulata, the performance of mitochondrial versus nuclear genes, and the impact of data partitions with heterogeneous base composition.  Syst. Biol. 48, 65-75 (1999).
Not used, as all trees presented are unrooted

262.	Springer, M. S., Burk, A., Kavanagh, J. R., Waddell, V. G. & Stanhope, M. J.  The interphotoreceptor retinoid binding protein gene in therian mammals: implications for higher level relationships and evidence for loss of function in the marsupial mole.  Proc. Natl. Acad. Sci. USA 94, 13754-13759 (1997).

263.	Springer, M. S. et al.  Endemic African mammals shake the phylogenetic tree. Nature 388, 61-64 (1997).

264.	Stanhope, M. J. et al. in Primates and Their Relatives in Phylogenetic Perspective (ed MacPhee, R. D. E.) 251-292  (Plenum, New York, 1993).

265.	Stanhope, M. J. et al.  Highly congruent molecular support for a diverse superordinal clade of endemic African mammals.  Mol. Phylogenet. Evol. 9, 501-508 (1998).

266.	Stanhope, M. J. et al.  Molecular evidence for multiple origins of Insectivora and for a new order of endemic African insectivore mammals.  Proc. Natl. Acad. Sci. USA 95, 9967-9972 (1998).

267.	Sterba, O.  Phylogenetic interpretation of the early development of the eutherian blastocyst.  Acta Veterin. Brno 65, 311-320 (1996).

268.	Stock, D. W., Quattro, J. M., Whitt, G. S. & Powers, D. A.  Lactate dehydrogenase (LDH) gene duplication during chordate evolution: the cDNA sequence of the LDH of the tunicate Styela plicata.  Mol. Biol. Evol. 14, 1273-1284 (1997).

269.	Sun, H. W. & Plapp, B. V.  Progressive sequence alignment and molecular evolution of the zinc containing alcohol dehydrogenase family.  J. Mol. Evol. 34, 522-535 (1992).

270.	Switzer, R. C., III, Johnson, J. I. & Kirsch, J. A. W.  Phylogeny through brain traits: relation of lateral olfactory tract fibers to the accessory olfactory formation as a palimpsest of mammalian descent.  Brain Behav. Evol. 17, 339-363 (1980).

271.	Szalay, F. S. & Lucas, S. G.  The postcranial morphology of Paleocene Chriacus and Mixodectes and the phylogenetic relationships of archontan mammals.  N. M. Mus. Nat. Hist. Sci. Bull. 7, 1-47 (1996).  
Not used, as no explicit tree is presented

272.	Takagi, T. et al.  Characterization and primary structure of Amphioxus troponin C. Eur. J. Biochem. 221, 537-546 (1994).

273.	Tan, D. P.  Cloning and sequence analysis of a cDNA encoding bovine ribosomal protein P2: predicted alpha-helices and potential phosphorylation sites.  DNA Seq. 7, 203-208 (1997).

274.	Tassy, P. & Shoshani, J. in The Phylogeny and Classification of the Tetrapods, Volume 2.  Mammals (ed Benton, M. J.) 283-315 (Clarendon, Oxford, 1988).

275.	Taylor, C. A. M., Coates, D. & Shirras, A. D.  The Acer gene of Drosophila codes for an angiotensin-converting enzyme homologue.  Gene 181, 191-197 (1996).

276.	Thewissen, J. G. M.  Phylogenetic aspects of cetacean origins: a morphological perspective.  J. Mammal. Evol. 2, 157-184 (1994).
Not used, as reference is a review of previous studies

277.	Thewissen, J. G. M. & Babcock, S. K.  Distinctive cranial and cervical innervation of wing muscles: new evidence for bat monophyly.  Science 251, 934-936 (1991).

278.	Thewissen, J. G. M. & Madar, S. I.  Ankle morphology of the earliest cetaceans and its implications for the phylogenetic relations among ungulates.  Syst. Biol. 48, 21-30 (1999).
Not used, as reference assumes an existing tree

279.	Thirstrup, K. et al.  Cloning and expression in insect cells of two pancreatic lipases and a procolipase from Myocastor coypus.  Eur. J. Biochem. 227, 186-193 (1995).

280.	Toyosawa, S., O'hUigin, C & Klein, J.  The dentin matrix protein 1 gene of prototherian and metatherian mammals.  J. Mol. Evol. 48, 160-167 (1999).

281.	Tsunoyama, K. & Gojobori, T.  Evolution of nicotinic acetylcholine receptor subunits.  Mol. Biol. Evol. 15, 518-527 (1998).

282.	Uchikawa, K.  Myobiid mites (Trombidiformes: Myobiidae) associated with the vampire bats (Chiroptera: Phyllostomatidae) and information on host taxonomy deduced from them.  J. Parasitol. 73, 640-645 (1987).
Not used, as no explicit tree is presented

283.	Uhlar, C. M. et al.  Wallaby serum amyloid A protein: cDNA cloning, sequence and evolutionary analysis.  Scand. J. Immunol. 43, 271-276 (1996).

284.	Valdenaire, O., Rohrbacher, E. & Mattei, M. G.  Organization of the gene encoding the human endothelin-converting enzyme (ECE-1).  J. Biol. Chem. 270, 29794-29798 (1995).

285.	van Bockxmeer, F. M. & Morgan, E. H.  Comparative aspects of transferrin-reticulocyte interactions: membrane receptors and iron uptake.  Comp. Biochem. Physiol. A 71, 211-218 (1982).

286.	Van Valen, L.  The evolution of bats.  Evol. Theor. 4, 103-121 (1979).

287.	van Wormhoudt, A. & Sellos, D.  Cloning and sequencing analysis of three amylase cDNAs in the shrimp Penaeus vannamei (Crustacea Decapoda): evolutionary aspects.  J. Mol. Evol. 42, 543-551 (1996).

288.	Vassilatis, D. K. et al.  Evolutionary relationship of the ligand-gated ion channels and the avermectin-sensitive, glutamate-gated chloride channels.  J. Mol. Evol. 44, 501-508 (1997).

289.	Ventura, M. M.  Phospholipases A-2: a dendrogram from a distance matrix based on size and hydrophobicity of the residues in their homologous sequences.  Anais Acad. Brasileira Cien. 62, 177-182 (1990).

290.	Vislobokova, I. A.  The main regularities of the historical development and classification of Ruminantia.  Paleo. Zhurnal 4, 3-14 (1990).
Not used, as tree presented is not explicitly phylogenetic

291.	von Ossowski, I., Hausner, G. & Loewen, P.  Molecular evolutionary analysis based on the amino acid sequence of catalase.  J. Mol. Evol. 37, 71-76 (1993).

292.	Vrana, P. B. & Wheeler, W. C.  Molecular evolution and phylogenetic utility of the polyubiquitin locus in mammals and higher vertebrates.  Mol. Phylogenet. Evol 6, 259-269 (1996).

293.	Vuillaumier, S., Kaltenboeck, B., Lecointre, G., Lehn, P. & Denamur, E. Phylogenetic analysis of cystic fibrosis transmembrane conductance regulator gene in mammalian species argues for the development of a rabbit model for cystic fibrosis.  Mol. Biol. Evol. 14, 372-380 (1997).

294.	Waddell, P. J., Cao, Y, Hauf, J. & Hasegawa, M.  Using novel phylogenetic methods to evaluate mammalian mtDNA, including amino acid-invariant sites-LogDet plus site stripping, to detect internal conflicts in the data, with special reference to the positions of hedgehog, armadillo, and elephant.  Syst. Biol. 48, 31-53 (1999).

295.	Waddell, P. J., Okada, N. & Hasegawa, M.  Towards resolving the interordinal relationships of placental mammals.  Syst. Biol. 48, 1-5 (1999).
Not used, as reference is a review of existing studies

296.	Wagstaff, P., Kang, H. Y., Mylott, D., Robbins, P. J. & White, M. K. Characterization of the avian GLUT1 glucose transporter: differential regulation of GLUT1 and GLUT3 in chicken embryo fibroblasts.  Mol. Biol. Cell 6, 1575-1589 (1995).

297.	Wahlert, J. H.  Skull morphologies and relationships of geomyoid rodents.  Am. Mus. Nov. 2812, 1-20 (1985).

298.	Wang, W. & Shakes, D. C.  Molecular evolution of the 14-3-3 protein family.  J. Mol. Evol. 43, 384-398 (1996).

299.	Watzinger, F., Mayr, B., Haring, E. & Lion, T.  High sequence similarity within ras exons 1 and 2 in different mammalian species and phylogenetic divergence of the ras gene family.  Mammal. Genome 9, 214-219 (1998).

300.	Wen, D. et al.  Erythropoietin structure-function relationships: high degree of sequence homology among mammals.  Blood 82, 1507-1516 (1993).

301.	Wettstein, P. J., Strausbauch, M., Johnston, S. L. & Slates, J.  The divergence of major histocompatibility complex class genes in Sciurus aberti.  Mol. Biol. Evol. 13, 56-66 (1996).

302.	Wible, J. R.  The eutherian stapedial artery: character analysis and implications for superordinal relationships.  Zool. J. Linn. Soc. 91, 107-136 (1987).
Not used, as reference assumes ordinal monophyly a priori

303.	Wible, J. R. & Novacek, M. J.  Cranial evidence for the monophyletic origin of bats.  Am. Mus. Nov. 2911, 1-19 (1988).

304.	Winefield, C. S., Farnden, K. J. F., Reynolds, P. H. S. & Marshall, C. J. Evolutionary analysis of aspartate aminotransferase.  J. Mol. Evol. 40, 455-463 (1995).

305.	Wolf, B., Reinecke, K., Aumann, K. D., Brigelius Flohe, R. & Flohe, L. Taxonomical classification of the guinea pig based on its copper/zinc superoxide dismutase sequence.  Biol. Chem. Hoppe-Seyler 374, 641-649 (1993).

306.	Woodburne, M. O. & Case, J. A.  Dispersal, vicariance, and the Late Cretaceous to Early Tertiary land mammal biogeography from South America to Australia.  J. Mammal. Evol. 3, 121-161 (1996).
Not used, as reference assumes an existing tree

307.	Wyss, A. R. et al.  South America's earliest rodent and recognition of a new interval of mammalian evolution.  Nature 365, 434-437 (1993).
Not used, as no explicit tree presented

308.	Wyss, A. R., Novacek, M. J. & McKenna, M. C.  Amino acid sequence versus morphological data and the interordinal relationships of mammals.  Mol. Biol. Evol. 4, 99-116 (1987).
Not used, as reference assumes ordinal monophyly a priori

309.	Xia, X.  The rate heterogeneity of nonsynonymous substitutions in mammalian mitochondrial genes.  Mol. Biol. Evol. 15, 336-344 (1998).
Not used, as reference assumes an existing tree, which is also unrooted

310.	Xue, H. Identification of major phylogenetic branches of inhibitory ligand-gated channel receptors.  J. Mol. Evol. 47, 323-333 (1998).

311.	Yokoyama, S. & Radlwimmer, F. B.  The 'five-sites' rule and the evolution of red and green color vision in mammals.  Mol. Biol. Evol. 15, 560-567 (1998).

312.	Yokoyama, S. & Yokoyama, R.  Adaptive evolution of photoreceptors and visual pigments in vertebrates.  Annu. Rev. Ecol. Syst. 27, 543-567 (1998).

313.	Yoshida, A., Rzhetsky, A., Hsu, L. C. & Chang, C.  Human aldehyde dehydrogenase gene family.  Eur. J. Biochem. 251, 549-557 (1998).

314.	Zeller, U., Wible, J. R. & Elsner, M.  New ontogenetic evidence on the septomaxilla of Tamandua and Choloepus (Mammalia, Xenarthra), with a reevaluation of the homology of the mammalian septomaxilla.  J. Mammal. Evol. 1, 31-46 (1993).
Not used, as reference assumes an existing tree

315.	Zietkiewicz, E. & Labuda, D.  Mosaic evolution of rodent B1 elements.  J. Mol. Evol. 42, 66-72 (1996).

Additional references

316.	Adkins, R. M., Gelke, E. L., Rowe, D. & Honeycutt, R. L. Molecular phylogeny and divergence time estimates for major rodent groups: Evidence from multiple genes. Molecular Biology and Evolution 18, 777-791 (2001).

317.	Adkins, R. M., Walton, A. H. & Honeycutt, R. L. Higher-level systematics of rodents and divergence time estimates based on two congruent nuclear genes. Molecular Phylogenetics and Evolution 26, 409-420 (2003).

318.	Aida, Y. et al. Cloning of Cdnas and the Molecular Evolution of a Bovine Mhc Class-Ii Dra Gene. Biochemical and Biophysical Research Communications 204, 195-202 (1994).

319.	Allard, M. W., Farris, J. S. & Carpenter, J. M. Congruence among Mammalian Mitochondrial Genes. Cladistics 15, 75-84 (1999).

320.	Allard, M. W., Miyamoto, M. M., Jarecki, L., Kraus, F. & Tennant, M. R. DNA systematics and evolution of the artiodactyl family Bovidae. Proceedings of the National Academy of Sciences of the United States of America 89, 3972-3976 (1992).

321.	Amrine-Madsen, H. et al. Nuclear gene sequences provide evidence for the monophyly of australidelphian marsupials. Molecular Phylogenetics and Evolution 28, 186-196 (2003).

322.	Amrine-Madsen, H., Koepfli, K.-P., Wayne, R. K. & Springer, M. S. A new phylogenetic marker, apolipoprotein B, provides compelling evidence for eutherian relationships. Molecular Phylogenetics and Evolution 28, 225-240 (2003).

323.	Arnason, U., Gullberg, A., Gretarsdottir, S., Ursing, B. & Janke, A. The mitochondrial genome of the sperm whale and a new molecular reference for estimating eutherian divergence dates. Journal of Molecular Evolution 50, 569-578 (2000).

324.	Arnason, U., Gullberg, A. & Janke, A. Mitogenomic analyses provide new insights into cetacean origin and evolution. Gene 333, 27-34 (2004).

325.	Arnason, U. & Janke, A. Mitogenomic analyses of eutherian relationships. Cytogenetics and Genome Research 96, 20-32 (2002).

326.	Arnason, U. et al. Mammalian mitogenomic relationships and the root of the eutherian tree. Proceedings of the National Academy of Sciences of the United States of America 99, 8151-8156 (2002).

327.	Asher, R. J. A morphological basis for assessing the phylogeny of the "Tenrecoidea" (Mammalia, Lipotyphla). Cladistics 15, 231-252 (1999).

328.	Asher, R. J. Phylogenetic history of tenrecs and other insectivoran mammals. (Ph.D. Diss., State University at Stony Brook, New York, 2000).

329.	Asher, R. J. Cranial anatomy in tenrecid insectivorans: character evolution across competing phylogenies. American Museum Novitates 3352, 1-54 (2001).

330.	Asher, R. J., McKenna, M. C., Emry, R. J., Tabrum, A. R. & Kron, D. G. Morphology and relationships of Apternodus and other extinct, zalambododont, placental mammals. Bulletin of the American Museum of Natural History 273, 1-117 (2002).

331.	Asher, R. J., Novacek, M. J. & Geisler, J. H. Relationships of endemic African mammals and their fossil relatives based on morphological and molecular evidence. Journal of Mammalian Evolution 10, 131-194 (2003).

332.	Aveskogh, M. & Hellman, L. Evidence for an early appearance of modern post-switch isotypes in mammalian evolution: Cloning of IgE, IgG and IgA from the marsupial Monodelphis domestica. European Journal of Immunology 28, 2738-2750 (1998).

333.	Bailey, W. J., Slighthom, J. L. & Goodman, M. Rejection of the "flying-primate" hypothesis by phylogenetic evidence from the epsilon-globin gene. Science 256, 86-89 (1992).

334.	Baker, M. L. et al. Further characterization of T cell receptor chains of marsupials. Developmental and Comparative Immunology 25, 495-507 (2001).

335.	Barros, M. C., Sampaio, I. & Schneider, H. Phylogenetic analysis of 16S mitochondrial DNA data in sloths and anteaters. Genetics and Molecular Biology 26, 5-11 (2003).

336.	Beintema, J. J., Breukelman, H. J., Dubois, J. Y. F. & Warmels, H. W. Phylogeny of ruminants secretory ribonuclease gene sequences of pronghorn (Antilocapra americana). Molecular Phylogenetics and Evolution 26, 18-25 (2003).

337.	Beintema, J. J. K. R. G. The ribonuclease A superfamily: general discussion. Cellular and Molecular Life Sciences 54, 8 (1998).

338.	Belov, K., Harrison, G. A., Miller, R. D. & Cooper, D. W. Molecular cloning of the brushtail possum (Trichosurus vulpecula) immunoglobulin E heavy chain constant region. Molecular Immunology 36, 1255-1261 (1999).

339.	Belov, K., Harrison, G. A., Rosenberg, G. H., Miller, R. D. & Cooper, D. W. Isolation and comparison of the IgM heavy chain constant regions from Australian (Trichosurus vulpecula) and American (Monodelphis domestica) marsupials. Developmental and Comparative Immunology 23, 649-656 (1999).

340.	Belov, K., Hellman, L. & Cooper, D. W. Characterization of immunoglobulin gamma 1 from a monotreme, Tachyglossus aculeatus. Immunogenetics 53, 1065-1071 (2002).

341.	Belov, K., Zenger, K. R., Hellman, L. & Cooper, D. W. Echidna IgA supports mammalian unity and traditional Therian relationship. Mammalian Genome 13, 656-663 (2002).

342.	Bentz, S. & Montgelard, C. Systematic position of the African dormouse Graphiurus (Rodentia, Gliridae) assessed from cytochrome b and 12S rRNA mitochondrial genes. Journal of Mammalian Evolution 6, 67-83 (1999).

343.	Breed, W. G., Hope, R. M., Wiebkin, O. W., Spargo, S. C. & Chapman, J. A. Structural organization and evolution of the marsupial zona pellucida. Reproduction 123, 13-21 (2002).

344.	Breukelman, H. J., van der Munnik, N., Kleineidam, R. G., Furia, A. & Beintema, J. J. Secretory ribonuclease genes and pseudogenes in true ruminants. Gene 212, 259-68 (1998).

345.	Breukelman, H. J. et al. Secretory ribonucleases in the primitive ruminant chevrotain (Tragulus javanicus). European Journal of Biochemistry 268, 3890-3897 (2001).

346.	Burk, A., Westerman, M., Kao, D. J., Kavanagh, J. R. & Springer, M. S. An analysis of marsupial interordinal relationships based on 12S rRNA, tRNA valine, 16S rRNA, and cytochrome b sequences. Journal of Mammalian Evolution 6, 317-334 (1999).

347.	Cao, Y., Adachi, J., Yano, T. A. & Hasegawa, M. Phylogenetic place of guinea-pigs - no support of the rodent-polyphyly hypothesis from maximum-likelihood analyses of multiple protein sequences. Molecular Biology and Evolution 11, 593-604 (1994).

348.	Cao, Y., Fujiwara, M., Nikaido, M., Okada, N. & Hasegawa, M. Interordinal relationships and timescale of eutherian evolution as inferred from mitochondrial genome data. Gene 259, 149-158 (2000).

349.	Catzeflis, F. M., Dickerman, A. W., Michaux, J. & Kirsch, J. A. W. in Mammal Phylogeny. Volume 2. Placentals. (eds. Szalay, F. S., Novacek, M. J. & McKenna, M. C.) (Springer Verlag, New York, 1993).

350.	Chang, B. S. W. & Campbell, D. L. Bias in phylogenetic reconstruction of vertebrate rhodopsin sequences. Molecular Biology and Evolution 17, 1220-1231 (2000).

351.	Conlon, J. M. The origin and evolution of peptide YY (PYY) and pancreatic polypeptide (PP). Peptides 23, 269-278 (2002).

352.	Conroy, C. J. & Cook, J. A. MtDNA evidence for repeated pulses of speciation within arvicoline and murid rodents. Journal of Mammalian Evolution 6, 221-245 (1999).

353.	Corneli, P. S. Complete Mitochondrial Genomes and Eutherian Evolution. Journal of Mammalian Evolution 9, 281-305 (2002).

354.	Cronin, J. E. & Sarich, V. M. in Comparative Biology and Evolutionary Relationships of Tree Shrews (ed. Luckett, W. P.) 293-312 (Plenum Press, New York, 1980).

355.	Cronin, M. A., Stuart, R., Pierson, B. J. & Patton, J. C. K-Casein gene phylogeny of higher ruminants (Pecora, Artiodactyla). Molecular Phylogenetics and Evolution 6, 295-311 (1996).

356.	Cui, S., Hope, R. M., Rathjen, J., Voyle, R. B. & Rathjen, P. D. Structure, sequence and function of a marsupial LIF gene: Conservation of IL-6 family cytokines. Cytogenetics and Cell Genetics 92, 271-278 (2001).

357.	Curlewis, J. D., Saunders, M. C., Kuang, J., Harrison, G. A. & Cooper, D. W. Cloning and sequence analysis of a pituitary prolactin cDNA from the brushtail possum (Trichosurus vulpecula). General and Comparative Endocrinology 111, 61-67 (1998).

358.	DeBry, R. W. Identifying conflicting signal in a multigene analysis reveals a highly resolved tree: the phylogeny of Rodentia (Mammalia). Systematic Biology 52, 604-617 (2003).

359.	DeBry, R. W. & Sagel, R. M. Phylogeny of Rodentia (Mammalia) inferred from the nuclear-encoded gene IRBP. Molecular Phylogenetics and Evolution 19, 290-301 (2001).

360.	de Jong, W. W. et al. Indels in protein-coding sequences of Euarchontoglires constrain the rooting of the eutherian tree. Molecular Phylogenetics and Evolution 28, 328-340 (2003).

361.	Delsuc, F., Catzeflis, F. M., Stanhope, M. J. & Douzery, E. J. P. The evolution of armadillos, anteaters and sloths depicted by nuclear and mitochondrial phylogenies: implications for the status of the enigmatic fossil Eurotamandua. Proceedings of the Royal Society Biological Sciences Series B 268, 1605-1615 (2001).

362.	Delsuc, F. et al. Molecular phylogeny of living xenarthrans and the impact of character and taxon sampling on the placental tree rooting. Molecular Biology and Evolution 19, 1656-1671 (2002). 

363.	Demmer, J., Stasiuk, S. J., Adamski, F. M. & Grigor, M. R. Cloning and expression of the transferrin and ferritin genes in a marsupial, the brushtail possum (Trichosurus vulpecula). Biochimica et Biophysica Acta 1445, 65-74 (1999).

364.	Demmer, J., Stasiuk, S. J., Grigor, M. R., Simpson, K. J. & Nicholas, K. R. Differential expression of the whey acidic protein gene during lactation in the brushtail possum (Trichosurus vulpecula). Biochimica et Biophysica Acta-Gene Struct. Expression 1522, 187-194 (2001).

365.	Douady, C. J. G. Molecular phylogenetics of the Insectivora (Ph.D. Diss., The Queen's University of Belfast, Belfast, 2001).

366.	Douady, C. J., Catzeflis, F, Kao, D. J., Springer, M. S. & Stanhope, M. J. Molecular evidence for the monophyly of Tenrecidae (Mammalia) and the timing of the colonization of Madagascar by Malagasy tenrecs. Molecular Phylogenetics and Evolution 22, 357-363 (2002).

367.	Douady, C. J., Catzeflis, F., Raman, J., Springer, M. S. & Stanhope, M. J. The Sahara as a vicariant agent, and the role of Miocene climatic events, in the diversification of the mammalian order Macroscelidea (elephant shrews). Proceedings of the National Academy of Sciences of the United States of America 100, 8325-8330 (2003).

368.	Douady, C. J. & Douzery, E. J. P. Molecular estimation of eulipotyphlan divergence times and the evolution of ''Insectivora ''. Molecular Phylogenetics and Evolution 28, 285-296 (2003).

369.	Douady, C J. et al. Molecular phylogenetic evidence confirming the Eulipotyphla concept and in support of hedgehogs as the sister group to shrews. Molecular Phylogenetics and Evolution 25, 200-209 (2002).

370.	Douady, C. J., Scally, M., Springer, M. S. & Stanhope, M. J. ''Lipotyphlan'' phylogeny based on the growth hormone receptor gene: a reanalysis. Molecular Phylogenetics and Evolution 30, 778-788 (2003).

371.	Dubois, J. Y. F., Ursing, B. M., Kolkman, J. A. & Beintema, J. J. Molecular evolution of mammalian ribonucleases 1. Molecular Phylogenetics and Evolution 27, 453-463 (2003).

372.	Duncan, R., Faggart, M. A. & Cornell, N. W. Phylogenetic analysis of the 5-aminilevulinate synthase gene. Biological Bulletin 193, 247-248 (1997).

373.	Duner, T. et al. Cloning, structural characterization and functional expression of a zebrafish bradykinin B2-related receptor. Biochemical Journal 364, 817-824 (2002).

374.	Emerson, G. L., Kilpatrick, C. W., McNiff, B. E., Ottenwalder, J. & Allard, M. W. Phylogenetic relationships of the order insectivora based on complete 12S rRNA sequences from mitochondria. Cladistics 15, 221-230 (1999).

375.	Fleming, M. A., Potter, J. D., Ramirez, C. J., Ostrander, G. K. & Ostrander, E. A. Understanding missense mutations in the BRCA1 gene: An evolutionary approach. Proceedings of the National Academy of Sciences of the United States of America 100, 1151-1156 (2003).

376.	Fujino, T., Navaratnam, N., Jarmuz, A., von Haeseler, A. & Scott, J. C-->U editing of apolipoprotein B mRNA in marsupials: identification and characterisation of APOBEC-1 from the American opossum Monodelphus domestica. Nucleic Acids Res 27, 2662-71 (1999).

377.	Gallardo, M. H. & Kirsch, J. A. W. Molecular relationships among Octodontidae (Mammalia: Rodentia: Caviomorpha). Journal of Mammalian Evolution 8, 73-89 (2001).

378.	Ganfornina, M. D., Gutierrez, G., Bastiani, M. & Sanchez, D. A Phylogenetic Analysis of the Lipocalin Protein Family. Molecular Biology and Evolution 17, 114-126 (2000).

379.	Gatesy, J., Matthee, C., DeSalle, R. & Hayashi, C. Resolution of a supertree/supermatrix paradox. Systematic Biology 51, 652-664 (2002).

380.	Gatesy, J., O'Grady, P. & Baker, R. H. Corroboration among data sets in simultaneous analysis: Hidden support for phylogenetic relationships among higher level artiodactyl taxa. Cladistics 15, 271-313 (1999).

381.	Gaucher, E. A. et al. The planetary biology of cytochrome P450 aromatases. BMC Biology 2, 1-35 (2004).

382.	Gaudin, T. J. Phylogenetic relationships among sloths (Mammalia, Xenarthra, Tardigrada): the craniodental evidence. Zoological Journal of the Linnean Society 140, 255-305 (2004).

383.	Geisler, J. H. & Uhen, M. D. Morphological support for a close relationship between hippos and whales. Journal of Vertebrate Paleontology 23, 991-996 (2003).

384.	Gemmell, N. J. & Westerman, M. Phylogenetic relationships within the Class Mammalia: a study using mitochondrial 12S RNA sequences. Journal of Mammalian Evolution 2, 3-23 (1994).

385.	Gonzalez, D. S. & Jordan, I. K. The a-Mannosidases: phylogeny and adaptive diversification. Molecular Biology and Evolution 17, 292-300 (2000).

386.	Goodman, M. in Phylogeny of Primates (eds. Luckett, W. P. & Szalay, F. S.) 219-248 (Plenum Press, New York, 1975).

387.	Goodman, M., Miyamoto, M. M. & Czelusniak, J. in Molecules and Morphology in Evolution: Conflict or Compromise? (ed. Patterson, C.) (Cambridge University Press, Cambridge, 1987).

388.	Harrison, G. A. et al. A survey of type I interferons from a marsupial and monotreme: implications for the evolution of the type I interferon gene family in mammals. Cytokine 21, 105-119 (2003).

389.	Hassanin, A. & Douzery, E. J. P. Molecular and morphological phylogenies of Ruminantia and the alternative position of the Moschidae. Systematic Biology 52, 206-228 (2003).

390.	Hassanin, A., Golub, R., Lewis, S. M. & Wu, G. E. Evolution of the recombination signal sequences in the Ig heavy-chain variable region locus of mammals. Proceedings of the National Academy of Sciences of the United States of America 97, 11415-11420 (2000).

391.	Honeycutt, R. L., Rowe, D. L. & Gallardo, M. H. Molecular systematics of the South American caviomorph rodents: relationships among species and genera in the family Octodontidae. Molecular Phylogenetics and Evolution 26, 476-489 (2003).

392.	Hooker, J. J. Tarsals of the extinct insectivoran family Nyctitheriidae (Mammalia): evidence for archontan relationships. Zoological Journal of the Linnean Society 132, 501-529 (2001).

393.	Hope, P. J. et al. Cloning of leptin cDNA and assignment to the long arm of chromosome 5 in the marsupial Sminthopsis crassicaudata. Cytogenetics and Cell Genetics 90, 22-29 (2000).

394.	Huchon, D., Catzeflis, F. M. & Douzery, E. J. P. Molecular evolution of the nuclear von Willebrand Factor gene in mammals and the phylogeny of rodents. Molecular Biology and Evolution 16, 577-589 (1999).

395.	Huchon, D., Catzeflis, F. M. & Douzery, E. J. P. Variance of molecular datings, evolution of rodents and the phylogenetic affinities between Ctenodactylidae and Hystricognathi. Proceedings of the Royal Society Biological Sciences Series B 267, 393-402 (2000).

396.	Huchon, D. et al. Rodent phylogeny and a timescale for the evolution of Glires: Evidence from an extensive taxon sampling using three nuclear genes. Molecular Biology and Evolution 19, 1053-1065 (2002). 

397.	Hudelot, C., Gowri-Shankar, V., Jow, H., Rattray, M. & Higgs, P. G. RNA-based phylogenetic methods: application to mammalian mitochondrial RNA sequences. Molecular Phylogenetics and Evolution 28, 241-252 (2003).

398.	Hughes, A. L. Evolutionary diversification of the mammalian defensins. CMLS Cellular and Molecular Life Sciences 56, 94-103 (1999).

399.	Hughes, A. L. Origin and evolution of viral interleukin-10 and other DNA virus genes with vertebrate homologues. Journal of Molecular Evolution 54, 90-101 (2002).

400.	Hughes, A. L. & Yeager, M. Molecular evolution of the vertebrate immune system. Bioessays 19, 777-786 (1997).

401.	Irwin, D. M. & Arnason, U. Cytochrome b gene of marine mammals: Phylogeny and evolution. Journal of Mammalian Evolution 2, 37-55 (1994).

402.	Janke, A., Magnell, O., Wieczorek, G., Westerman, M. & Arnason, U. Phylogenetic analysis of 18S rRNA and the mitochondrial genomes of the wombat, Vombatus ursinus, and the spiny anteater, Tachyglossus aculeatus: Increased support for the Marsupionta hypothesis. Journal of Molecular Evolution 54, 71-80 (2002).

403.	Jansa, S. A. & Voss, R. S. Phylogenetic studies on didelphid marsupials I. Introduction and preliminary results from nuclear IRBP gene sequences. Journal of Mammalian Evolution 7, 43-77 (2000).

404.	Jow, H., Hudelot, C., Rattray, M. & Higgs, P. G. Bayesian phylogenetics using an RNA substitution model applied to early mammalian evolution. Molecular Biology and Evolution 19, 1591-1601 (2002).

405.	Killian, J. K., Buckley, T. R., Stewart, N., Munday, B. L. & Jirtle, R. L. Marsupials and eutherians reunited: Genetic evidence for the Theria hypothesis of mammalian evolution. Mammalian Genome 12, 513-517 (2001).

406.	Kirsch, J. A. W., Hutcheon, J. M., Byrnes, D. G. P. & Lloyd, B. D. Affinities and historical zoogeography of the New Zealand short-tailed bat, Mystacina tuberculata Gray 1843, inferred from DNA-hybridization comparisons. Journal of Mammalian Evolution 5, 33-64 (1998).

407.	Kirsch, J. A. W. & Mayer, G. C. The platypus is not a rodent: DNA hybridization, amniote phylogeny and the palimpsest theory. Philosophical Transactions of the Royal Society of London B Biological Sciences 353, 1221-1237 (1998).

408.	Kleineidam, R. G., Pesole, G., Breukelman, H. J., Beintema, J. J. & Kastelein, R. A. Inclusion of cetaceans within the order Artiodactyla based on phylogenetic analysis of pancreatic ribonuclease genes. Journal of Molecular Evolution 48, 360-368 (1999).

409.	Klonisch, T., Froehlich, C., Tetens, F., Fischer, B. & Hombach-Klonisch, S. Molecular remodeling of members of the relaxin family during primate evolution. Molecular Biology and Evolution 18, 393-403 (2001).

410.	Koehler, N., Gallardo, M., Contreras, L. & Torres-Mura, J. Allozymic variation and systematic relationships of the Octodontidae and allied taxa (Mammalia, Rodentia). Journal of Zoology 252, 243-250 (2000).

411.	Kupfermann, H., Satta, Y., Takahata, N., Tichy, H. & Klein, J. Evolution of Mhc-DRB introns: Implications for the origin of primates. Journal of Molecular Evolution 48, 663-674 (1999).

412.	Lam, M. K. P., Belov, K., Harrison, G. A. & Cooper, D. W. Cloning of the MHC class II DRB cDNA from the brushtail possum (Trichosurus vulpecula). Immunology Letters 76, 31-36 (2001).

413.	Langer, P. Evidence from the digestive tract on phylogenetic relationships in ungulates and whales. Journal of Zoological Systematics and Evolutionary Research 39, 77-90 (2001).

414.	Lee, M. H., Shroff, R., Cooper, S. J. B. & Hope, R. Evolution and molecular characterization of a beta-globin gene from the Australian echidna Tachyglossus aculeatus (Monotremata). Molecular Phylogenetics and Evolution 12, 205-214 (1999).

415.	Lemelin, P. Micro-anatomy of the volar skin and interordinal relationships of primates. Journal of Human Evolution 38, 257-267 (2000).

416.	Lin, Y. H. et al. Four new mitochondrial genomes and the increased stability of evolutionary trees of mammals from improved taxon sampling. Molecular Biology and Evolution 19, 2060-2070 (2002).

417.	Lin, Y. H. & Penny, D. Implications for bat evolution from two new complete mitochondrial genomes. Molecular Biology and Evolution 18, 684-688 (2001).

418.	Lucero, J. E., Rosenberg, G. H. & Miller, R. D. Marsupial light chains: Complexity and conservation of lambda in the opossum Monodelphis domestica. Journal of Immunology 161, 6724-6732 (1998).

419.	Ma, D.-P., Zharkikh, A., Graur, D., Vandeberg, J. L. & Li, W.-H. Structure and Evolution of Opposum, Guinea Pig and Porcupine Cytochrome b Genes. Journal of Molecular Evolution 36 (1993).

420.	Madsen, O. et al. Parallel adaptive radiations in two major clades of placental mammals. Nature 409, 610-614 (2001).

421.	Madsen, O., Willemsen, D., Ursing, B. M., Arnason, U. & de Jong, W. W. Molecular evolution of the mammalian alpha 2B adrenergic receptor. Molecular Biology and Evolution 19, 2150-2160 (2002).

422.	Malia, M. J., Adkins, R. M. & Allard, M. W. Molecular support for Afrotheria and the polyphyly of Lipotyphla based on analyses of the growth hormone receptor gene. Molecular Phylogenetics and Evolution 24, 91-101 (2002).

423.	Malia, M. J., Lipscomb, D. L. & Allard, M. W. The misleading effects of composite taxa in supermatrices. Molecular Phylogenetics and Evolution 27, 522-527 (2003).

424.	Marivaux, L., Vianey-Liaud, M. & Jaeger, J.-J. High-level phylogeny of early Tertiary rodents: dental evidence. Zoological Journal of the Linnean Society 142, 105-134 (2004).

425.	Mason, R. W. et al. Evolution of placental proteases. Biological Chemistry 383, 1113-1118 (2002).

426.	Maston, G. A. & Ruvolo, M. Chorionic gonadotropin has a recent origin within primates and an evolutionary history of selection. Molecular Biology and Evolution 19, 320-335 (2002).

427.	Mathur, A. K. & Polly, P. D. The evolution of enamel microstructure: How important is amelogenin? Journal of Mammalian Evolution 7, 23-42 (2000).

428.	Matthee, C. A., Burzlaff, J. D., Taylor, J. F. & K., D. S. Mining the mammalian genome for artiodactyl systematics. Systematic Biology 50, 367-390 (2001).

429.	McNiff, B. E & Allard, M. W. A test of Archonta monophyly and the phylogenetic utility of the mitochondrial gene 12S rRNA. American Journal of Physical Anthropology 107, 225-241 (1998).

430.	Meng, J., Hu, Y. M. & Li, C. K. The osteology of Rhombomylus (Mammalia, Glires): Implications for phylogeny and evolution of Glires. Bulletin of the American Museum of Natural History 275, 1-247 (2003).

431.	Michaux, J. & Catzeflis, F. The Bushlike Radiation of Muroid Rodents Is Exemplified by the Molecular Phylogeny of the LCAT Nuclear Gene. Molecular Phylogenetics and Evolution 17, 280-293 (2000).

432.	Mindell, D. P. et al. Interordinal relationships of birds and other reptiles based on whole mitochondrial genomes. Systematic Biology 48, 138-152 (1999).

433.	Misawa, K. & Janke, A. Revisiting the Glires concept —phylogenetic analysis of nuclear sequences. Molecular Phylogenetics and Evolution 28, 320-327 (2003).

434.	Miska, K.-B. & Miller, R.-D. Marsupial Mhc class I: classical sequences from the opossum, Monodelphis domestica. Immunogenetics 50, 89-93 (1999).

435.	Miyamoto, M. M., Porter, C. A. & Goodman, M. c-myc gene sequences and the phylogeny of bats and other eutherian mammals. Systematic Biology 49, 501-514 (2000).

436.	Mouchaty, S. K., Catzeflis, F., Janke, A. & Arnason, U. Molecular evidence of an African Phiomorpha-South American Caviomorpha clade and support for Hystricognathi based on the complete mitochondrial genome of the cane rat (Thryonomys swinderianus). Molecular Phylogenetics and Evolution 18, 127-135 (2001).

437.	Mouchaty, S. K., Gullberg, A., Janke, A. & Arnason, U. The phylogenetic position of the Talpidae within Eutheria based on analysis of complete mitochondrial sequences. Molecular Biology and Evolution 17, 60-67 (2000).

438.	Murata, Y. et al. Afrotherian phylogeny as inferred from complete mitochondrial genomes. Molecular Phylogenetics and Evolution 28, 253-260 (2003).

439.	Murphy, W. J. et al. Molecular phylogenetics and the origins of placental mammals. Nature 409, 614-618 (2001).

440.	Murphy, W. J. et al. Resolution of the early placental mammal radiation using Bayesian phylogenetics. Science 294, 2348-2351 (2001).

441.	Nagai, K. Molecular evolution of Sry and Sox gene. Gene 270, 161-169 (2001).

442.	Narita, Y., Oda, S., Takenaka, O. & Kageyama, T. Phylogenetic position of Eulipotyphla inferred from the cDNA sequences of pepsinogens A and C. Molecular Phylogenetics and Evolution 21, 32-42 (2001).

443.	Nei, M. & Glazko, G. V. Estimation of divergence times for a few mammalian and several primate species. Journal of Heredity 93, 157-164 (2002).

444.	Nijman, I. J., van Tessel, P. & Lenstra, J. A. SINE retrotransposition during the evolution of the pecoran ruminants. Journal of Molecular Evolution 54, 9-16 (2002).

445.	Nikaido, M., Harada, M., Cao, Y., Hasegawa, M. & Okada, N. Monophyletic origin of the order Chiroptera and its phylogenetic position among Mammalia, as inferred from the complete sequence of the mitochondrial DNA of a Japanese megabat, the Ryukyu flying fox (Pteropus dasymallus). Journal of Molecular Evolution 51, 318-328 (2000).

446.	Nikaido, M. et al. Maximum likelihood analysis of the complete mitochondrial genomes of eutherians and a reevaluation of the phylogeny of bats and insectivores. Journal of Molecular Evolution 53, 508-516 (2001).

447.	Nikaido, M., Cao, Y., Harada, M., Okada, N. & Hasegawa, M. Mitochondrial phylogeny of hedgehogs and monophyly of Eulipotyphla. Molecular Phylogenetics and Evolution 28, 276-284 (2003).

448.	Nishihara, H., Terai, Y. & Okada, N. Characterization of Novel Alu- and tRNA-Related SINEs from the Tree Shrew and Evolutionary Implications of Their Origins. Molecular Biology and Evolution 19, 1964-1972 (2002).

449.	Novacek, M. J. in Comparative Biology and Evolutionary Relationships of Tree Shrews (ed. Luckett, W. P.) 35-93 (Plenum Press, New York, 1980).

450.	Old, J. M., Deane, E. M. & Harrison, G. A. Molecular characterisation of the tammar wallaby (Macropus eugenii) CD3 epsilon chain cDNA. Molecular Immunology 38, 359-364 (2001).

451.	O'Leary, M. A. Parsimony analysis of total evidence from extinct and extant taxa and the cetacean–artiodactyl question (Mammalia, Ungulata). Cladistics 15, 315-330 (1999).

452.	O'Leary, M. A. & Geisler, J. H. The position of Cetacea within Mammalia: Phylogenetic analysis of morphological data from extinct and extant taxa. Systematic Biology 48, 455-490 (1999).

453.	Onuma, M., Cao, Y., Hasegawa, M. & Kusakabe, S. A close relationship of Chiroptera with Eulipotyphla (core Insectivora) suggested by four mitochondrial genes. Zoological Science Tokyo 17, 1327-1332 (2000).

454.	Onuma, M., Kusakabe, T. & Kusakabe, S. Phylogenetic Positions of Insectivora in Eutheria Inferred from Mitochondrial Cytochrome c Oxidase Subunit II Gene. Zoological Science 15, 139-145 (1998).

455.	Penny, D., Hasegawa, M., Waddell, P. J. & Hendy, M. D. Mammalian evolution: Timing and implications from using the LogDeterminant transform for proteins of differing amino acid composition. Systematic Biology 48, 76-93 (1999).

456.	Philippe, H. & Douzery, E. The pitfalls of molecular phlyogeny based on four species, as illustrated by the Cetacea/Artiodactyla relationships. Journal of Mammalian Evolution 2, 133-152 (1994).

457.	Piotte, C. P., Hunter, A. K., Marshall, C. J. & Grigor, M. R. Phylogenetic analysis of three lipocalin-like proteins present in the milk of Trichosurus vulpecula (Phalangeridae, Marsupialia). Journal of Molecular Evolution 46, 361-369 (1998).

458.	Pitra, C. & Veits, J. Use of mitochondrial DNA sequences to test the Ceratomorpha (Perissodactyla: Mammalia) hypothesis. Journal of Zoological Systematics and Evolutionary Research 38, 65-72 (2000).

459.	Plouhinec, J. L. et al. The mammalian Crx genes are highly divergent representatives of the Otx5 gene family, a gnathostome orthology class of orthodenticle-related homeogenes involved in the differentiation of retinal photoreceptors and circadian entrainment. Mol Biol Evol 20, 513-21 (2003).

460.	Porter, C. A., Goodman, M. & Stanhope, M. J. Evidence on mammalian phylogeny from sequences of exon 28 of the von Willebrand Factor gene. Molecular Phylogenetics and Evolution 5, 89-101 (1996).

461.	Power, D. M. et al. Evolution of the thyroid hormone-binding protein, transthyretin. General and Comparative Endocrinology 119, 241-255 (2000).

462.	Prapunpoj, P., Richardson, S. J., Fumagalli, L. & Schreiber, G. The evolution of the thyroid hormone distributor protein transthyretin in the order Insectivora, class Mammalia. Molecular Biology and Evolution 17, 1199-1209 (2000).

463.	Randi, E., Lucchini, V. & Diong, C. H. Evolutionary genetics of the Suiformes as reconstructed using mtDNA sequences. Journal of Mammalian Evolution 3, 163-194 (1996).

464.	Randi, E., Mucci, N., Pierpaoli, M. & Douzery, E. New phylogenetic perspectives on the Cervidae (Artiodactyla) are provided by the mitochondrial cytochrome b gene. Proceedings of the Royal Society of London Series B-Biological Sciences 265, 793-801 (1998).

465.	Reyes, A. et al. Congruent mammalian trees from mitochondrial and nuclear genes using Bayesian methods. Molecular Biology and Evolution 21, 397-403 (2004).

466.	Richards, M. H. & Nelson, J. L. The evolution of vertebrate antigen receptors: A phylogenetic approach. Molecular Biology and Evolution 17, 146-155 (2000).

467.	Rijnkels, M. Multispecies comparison of the casein gene loci and evolution of casein gene family. Journal of Mammary Gland Biology and Neoplasia 7, 327-345 (2002).

468.	Robinson-Rechavi, M., Ponger, L. & Mouchiroud, D. Nuclear gene LCAT supports rodent monophyly. Molecular Biology and Evolution 17, 1410-1412 (2000).

469.	Roca, A. L. et al. Mesozoic origin for West Indian insectivores. Nature 429, 649-651 (2004).

470.	Rowe, D. L. & Honeycutt, R. L. Phylogenetic relationships, ecological correlates, and molecular evolution within the Cavioidea (Mammalia, Rodentia). Molecular Biology and Evolution 19, 263-277 (2002).

471.	Salaneck, E., Fredriksson, R., Larson, E. T., Conlon, J. M. & Larhammar, D. A neuropeptide Y receptor Y1-subfamily gene from an agnathan, the European river lamprey - A potential ancestral gene. European Journal of Biochemistry 268, 6146-6154 (2001).

472.	Sarich, V. M. in Mammal Phylogeny. Volume 2. Placentals. (eds. Szalay, F. S., Novacek, M. J. & McKenna, M. C.) 103-114 (Springer-Verlag, New York, 1993).

473.	Scally, M. et al. Molecular evidence for the major clades of placental mammals. Journal of Mammalian Evolution 8, 239-276 (2001).

474.	Schmitz, A., Ohme, M., Suryobroto, B. & Zischler, H. The colugo (Cynocephalus variegatus, Dermoptera): The primates' gliding sister? Molecular Biology and Evolution 19, 2308-2312 (2002).

475.	Schmitz, J. & Zischler, H. A novel family of tRNA-derived SINEs in the colugo and two new retrotransposable markers separating dermopterans from primates. Molecular Phylogenetics and Evolution 28, 341-349 (2003).

476.	Scott, K. M. & Janis, C. M. in Mammal Phylogeny. Volume 2. Placentals. (eds. Szalay, F. S., Novacek, M. J. & McKenna, M. C.) 282-302 (Springer-Verlag, New York, 1993).

477.	Serdobova, I. M. & Kramerov, D. A. Short retroposons of the B2 superfamily: Evolution and application for the study of rodent phylogeny. Journal of Molecular Evolution 46, 202-214 (1998).

478.	Seyfert, H.-M. et al. Molecular characterization of STAT5A- and STAT5B-encoding genes reveals extended intragenic sequence homogeneity in cattle and mouse and different degrees of divergent evolution of various domains. Journal of Molecular Evolution 50, 550-561 (2000).

479.	Shevchuk, N. A. & Allard, M. W. Sources of incongruence among mammalian mitochondrial sequences: COII, COIII, and ND6 genes are main contributors. Molecular Phylogenetics and Evolution 21, 43-54 (2001).

480.	Spargo, S. C. & Hope, R. M. Evolution and nomenclature of the Zona Pellucida gene family. Biology of Reproduction 68, 358-362 (2003).

481.	Springer, M. S. & Kirsch, J. A. A molecular perspective on the phylogeny of placental mammals based on mitochondrial 12S rRNA sequences, with special reference to problems of the Paenungulata. Journal of Mammalian Evolution 1, 149-166 (1993).

482.	Springer, M. S., Teeling, E. C., Madsen, O., Stanhope, M. J. & de Jong, W. W. Integrated fossil and molecular data reconstruct bat echolocation. Proceedings of the National Academy of Sciences of the United States of America 98, 6241-6246 (2001).

483.	Stanhope, M. J. et al. Mammalian evolution and the interphotoreceptor retinoid binding protein (IRBP) gene: Convincing evidence for several superordinal clades. Journal of Molecular Evolution 43, 83-92 (1996).

484.	Stasiuk, S. J., Summers, E. L. & Demmer, J. Cloning of a marsupial kappa-casein cDNA from the brushtail possum (Trichosurus vulpecula). Reproduction Fertility and Development 12, 215-222 (2000).

485.	Stock, D. W. & Powers, D. A. The cDNA sequence of the lactate dehydrogenase-A of the spiny dogfish (Squalus acanthias): Corrections to the amino, acid sequence and an analysis of the phylogeny of vertebrate lactate dehydrogenases. Molecular Marine Biology and Biotechnology 4, 284-294 (1995).

486.	Stock, D. W. & Powers, D. A. A monophyletic origin of heart-predominant lactate dehydrogenase (LDH) isozymes of gnathostome vertebrates: Evidence from the cDNA sequence of the spiny dogfish (Squalus acanthias) LDH-B. Molecular Marine Biology and Biotechnology 7, 160-164 (1998).

487.	Stock, D. W. & Whitt, G. S. Evolutionary Implications of the cDNA sequence of the single lactate dehydrogenase of a lamprey. Proceedings of the National Academy of Sciences of the United States of America 89, 1799-1803 (1992).

488.	Stone, W. H. et al. Identification and sequence analysis of an Mhc class II B gene in a marsupial (Monodelphis domestica). Immunogenetics 49, 461-463 (1999).

489.	Su, C., Nguyen, V. K. & Nei, M. Adaptive evolution of variable region genes encoding an unusual type of immunoglobulin in camelids. Molecular Biology and Evolution 19, 205-215 (2002).

490.	Takahashi, K., Rooney, A. P. & Nei, M. Origins and divergence times of mammalian class II MHC gene clusters. Journal of Heredity 91, 198-204 (2000).
 
491.	Taylor, C. L., Harrison, G. A., Watson, C. M. & Deane, E. M. cDNA cloning of the polymeric immunoglobulin receptor of the marsupial Macropus eugenii (tammar wallaby). European Journal of Immunogenetics 29, 87-93 (2002).

492.	Teeling, E. C. et al. Molecular evidence regarding the origin of echolocation and flight in bats. Nature 403, 188-192 (2000).

493.	Teeling, E. C. et al. Microbat paraphyly and the convergent evolution of a key innovation in Old World rhinolophoid microbats. Proceedings of the National Academy of Sciences of the United States of America 99, 1431-1436 (2002).

494.	Teeling, E. C., Madsen, O., Murphy, W. J., Springer, M. S. & O'Brien, S. J. Nuclear gene sequences confirm an ancient link between New Zealand's short-tailed bat and South American noctilionoid bats. Mol Phylogenet Evol 28, 308-19 (2003).

495.	Thewissen, J. G. M. & Babcock, S. K. in Primates and their Relatives in Phylogenetic Perspective (ed. MacPhee, R. D. E.) 91-110 (Plenum, New York, 1993).

496.	Thewissen, J., Williams, E., Roe, L. & Hussain, S. Skeletons of terrestrial cetaceans and the relationship of whales to artiodactyls. Nature 413, 277-281 (2001).

497.	Toyosawa, S., O, H. C., Figueroa, F., Tichy, H. & Klein, J. Identification and characterization of amelogenin genes in monotremes, reptiles, and amphibians. Proceedings of the National Academy of Sciences of the United States of America 95, 13056-13061 (1998).

498.	Van Den Bussche, R. A., Baker, R. J., Huelsenbeck, J. P. & Hillis, D. M. Base compositional bias and phylogenetic analyses: A test of the "flying DNA" hypothesis. Molecular Phylogenetics and Evolution 10, 408-416 (1998).

499.	Van Den Bussche, R. A., Hoofer, S. R. & Hansen, E. W. Characterization and phylogenetic utility of the mammalian protamine P1 gene. Molecular Phylogenetics and Evolution 22, 333-341 (2002).

500.	van Dijk, M. A. M. et al. Protein sequence signatures support the African clade of mammals. Proceedings of the National Academy of Sciences of the United States of America 98, 188-193 (2001).

501.	van Dijk, M. A. M., Paradis, E., Catzeflis, F. & de Jong, W. W. The virtues of gaps: Xenarthran (Edentate) monophyly supported by a unique deletion in alpha A-crystallin. Systematic Biology 48, 94-106 (1999).

502.	van Rheede, T., Amons, R., Stewart, N. & de Jong, W. W. Lactate Dehydrogenase A as a highly abundant eye lens protein in platypus (Ornithorhynchus anatinus ):Upsilon (t -Crystallin. Molecular Biology and Evolution 20, 994-998 (2003).

503.	van Rheede, T., Smolenaars, M. M. W., Madsen, O. & de Jong, W. W. Molecular evolution of the mammalian prion protein. Molecular Biology and Evolution 20, 111-121 (2003).

504.	Vassetzky, N. S., Ten, O. A. & Kramerov, D. A. B1 and related SINEs in mammalian genomes. Gene 319, 149-160 (2003).

505.	Vernersson, M., Aveskogh, M., Munday, B. & Hellman, L. Evidence for an early appearance of modern post-switch immunoglobulin isotypes in mammalian evolution (II); cloning of IgE, IgG1 and IgG2 from a monotreme, the duck-billed platypus, Omithorhynchus anatinus. European Journal of Immunology 32, 2145-2155 (2002).

506.	Waddell, P. J., Cao, Y., Hasegawa, M. & Mindell, D. P. Assessing the Cretaceous superordinal divergence times within birds and placental mammals by using whole mitochondrial protein sequences and an extended statistical framework. Systematic Biology 48, 119-137 (1999).

507.	Waddell, P. J., Kishino, H. & Ota, R. A phylogenetic foundation for comparative mammalian genomics. Genome Informatics 12, 141-154 (2001).

508.	Waddell, P. J. & Shelley, S. Evaluating placental inter-ordinal phylogenies with novel sequences including RAG1, gamma-fibrinogen, ND6, and mt-tRNA, plus MCMC-driven nucleotide, amino acid, and codon models. Molecular Phylogenetics and Evolution 28, 197-224 (2003).

509.	Whitworth, D. J. et al. Characterization of steroidogenic factor 1 during sexual differentiation in a marsupial. Gene 277, 209-219 (2001).

510.	Wu, W., Schmidt, T. R., Goodman, M. & Grossman, L. I. Molecular evolution of cytochrome c oxidase subunit I in primates: Is there coevolution between mitochondrial and nuclear genomes? Molecular Phylogenetics and Evolution 17, 294-304 (2000).

511.	Xia, X., Xie, Z. & Kjer, K. M. 18S ribosomal RNA and tetrapod phylogeny. Syst Biol 52, 283-95 (2003).

512.	Yoder, A. D. et al. Single origin of Malagasy Carnivora from an African ancestor. Nature 421, 734-737 (2003).

513.	Yokoyama, S. Gene duplications and evolution of the short wavelength- sensitive visual pigments in vertebrates. Molecular Biology and Evolution 11, 32-39 (1994).

514.	Yokoyama, S. Amino-acid replacements and wavelength absorption of visual pigments in vertebrates. Molecular Biology and Evolution 12, 53-61 (1995).

515.	Yokayama, S. & Radlwimmer, F. B. The "five-sites" rule and the evolution of red and green color vision in mammals. Molecular Biology and Evolution 15, 560-567 (1998).

516.	Zhang, J. & Rosenberg, H. F. Diversifying selection of the tumor-growth promoter Angiogenin in primate evolution. Molecular Biology and Evolution 19, 438-445 (2002).

517.	Zhou, R. J., Guo, Y. Q., Cheng, H. H., Mao, Y. & Yu, Q. X. Comparative tree of sex-determining region Y (SRY) and SRY box genes. Acta Zoologica Sinica 43, 192-196 (1997).

518.	Zolzer, U. & von-Hagen, H. Amino acid sequences of P1 protamines and the phylogeny of eutherian mammals: a cladistic study. Comparative Biochemistry and Physiology B Biochemistry and Molecular Biology 110B, 805-815 (1995).

519.	Zuccolotto, P. D., Harrison, G. A. & Deane, E. M. Cloning of marsupial T cell receptor alpha and beta constant region cDNAs. Immunology and Cell Biology 78, 103-109 (2000).
